# Alternative Computational Protocols for Supercharging Protein Surfaces for Reversible Unfolding and Retention of Stability

**DOI:** 10.1371/journal.pone.0064363

**Published:** 2013-05-31

**Authors:** Bryan S. Der, Christien Kluwe, Aleksandr E. Miklos, Ron Jacak, Sergey Lyskov, Jeffrey J. Gray, George Georgiou, Andrew D. Ellington, Brian Kuhlman

**Affiliations:** 1 Department of Biochemistry and Biophysics, University of North Carolina at Chapel Hill, Chapel Hill, North Carolina, United States of America; 2 Center for Systems and Synthetic Biology, University of Texas at Austin, Austin, Texas, United States of America; 3 Institute for Cellular and Molecular Biology, University of Texas at Austin, Austin, Texas, United States of America; 4 Applied Research Laboratories, University of Texas at Austin, Austin, Texas, United States of America; 5 Department of Chemical and Biomolecular Engineering, Johns Hopkins University, Baltimore, Maryland, United States of America; 6 Lineberger Comprehensive Cancer Center, University of North Carolina at Chapel Hill, Chapel Hill, North Carolina, United States of America; Wake Forest University, United States of America

## Abstract

Reengineering protein surfaces to exhibit high net charge, referred to as “supercharging”, can improve reversibility of unfolding by preventing aggregation of partially unfolded states. Incorporation of charged side chains should be optimized while considering structural and energetic consequences, as numerous mutations and accumulation of like-charges can also destabilize the native state. A previously demonstrated approach deterministically mutates flexible polar residues (amino acids DERKNQ) with the fewest average neighboring atoms per side chain atom (AvNAPSA). Our approach uses Rosetta-based energy calculations to choose the surface mutations. Both protocols are available for use through the ROSIE web server. The automated Rosetta and AvNAPSA approaches for supercharging choose dissimilar mutations, raising an interesting division in surface charging strategy. Rosetta-supercharged variants of GFP (RscG) ranging from −11 to −61 and +7 to +58 were experimentally tested, and for comparison, we re-tested the previously developed AvNAPSA-supercharged variants of GFP (AscG) with +36 and −30 net charge. Mid-charge variants demonstrated ∼3-fold improvement in refolding with retention of stability. However, as we pushed to higher net charges, expression and soluble yield decreased, indicating that net charge or mutational load may be limiting factors. Interestingly, the two different approaches resulted in GFP variants with similar refolding properties. Our results show that there are multiple sets of residues that can be mutated to successfully supercharge a protein, and combining alternative supercharge protocols with experimental testing can be an effective approach for charge-based improvement to refolding.

## Introduction

Reengineering protein surfaces to have increased net charge can prevent ordered and disordered aggregation of partially unfolded states [1], [2]. Charge repulsion interactions disfavor two or more proteins coming into close proximity and subsequently aggregating via specific [3], [4], [5], [6] or non-specific [7] interactions. Net charge, rather than number of charged residues, is a major determinant of aggregation propensity [8], [9], and “supercharging” proteins to have increased net charge can thus prevent aggregation and promote appropriate refolding.

Aggregation is a common obstacle for protein applications in biotechnology and medicine. In medicine, preventing aggregation can improve the consistency and bioavailability of therapeutics, facilitate production and storage, safeguard drug activity, and curb immunogenicity [10]. Methods for inhibiting protein aggregation have been highly sought to improve biopharmaceuticals, from rational design to introduction of excipients [11], [12], [13]. For example, human calcitonin is a small peptide hormone required for calcium regulation and bone formation that is prone to forming amyloid fibrils. Calcitonin was redesigned with several mutations to arginine and lysine, and the resulting variant showed significantly reduced aggregation propensity and maintained/improved potency [14].

In biotechnology, sequestration of poorly soluble or readily misfolded proteins into inclusion-bodies is a bottleneck for expression and purification [15]. Enteropeptidase light chain cleaves trypsinogen into active trypsin and is used in various biotechnology applications, but it has poor solubility and refolding properties. Recent work by Simeonov *et al.* demonstrated that five mutations increasing the net charge from −3 to −9 resulted in improved in solubility and refolding yield without affecting structure or activity [16], [17]. Increasing net charge using surface mutations can also improve refolding of more complex proteins with limited plasticity, such as antibodies. While single-chain variable fragment antibodies (scF_V_s) have diverse applications, they show a tendency to aggregate upon unfolding [18]. Refrigeration is a necessary complication for storage, and even brief exposures to high temperature may cause irreversible unfolding of the scF_V_. Lyophilization is commonly used for long-term storage of proteins though this does not prevent aggregation upon rehydration [19]. Our previous work in supercharging scF_V_s demonstrates that after exposure to high temperature, a supercharged scF_V_ variant refolds and retains epitope binding, in contrast to the wild type parent [20].

Apart from promoting refolding, there are additional motivations for adding charges to protein surfaces. In the context of viral pathogenesis, it was discovered that highly cationic proteins and peptides are capable of facilitating cellular uptake [21]. While multiple groups have examined ‘natural’ cationic proteins such as HIV-Tat and antennapedia, others have employed ‘arginine-grafting’ as an approach to impart this function [22]. There is great interest in this field as protein-based nonviral cell entry can mediate intracellular delivery of therapeutic and antimicrobial biologics [23], [24], [25], [26], [27].

Additionally, engineering proteins to alleviate aggregation may lead to improved understanding of aggregation mechanisms and development of new strategies to prevent and treat diseases caused by protein aggregation [28]. Amyloid, prion, polyglutamine, and sickle-cell are aggregation-based diseases (reviewed in [29]). Recently, a study by Xu *et al.* implicated aggregation of p53 mutants in uncontrolled cell growth, and mutation of an isoleucine to arginine helped offset aggregation [30].

Adding charge to proteins can prevent aggregation, but it can also destabilize the folded state. Choosing which residues to mutate while retaining the native structure is a critical step in supercharging proteins. One approach explored by the David Liu group mutates the most highly solvent-exposed flexible polar residues, assuming that these positions will be able to accommodate any charged side chain [31]. This method, called AvNAPSA (Average number of Neighboring Atoms Per Side-chain Atom) has been used successfully in some cases. For example, variants of sfGFP, streptavidin, and glutathione S-transferase demonstrated improved solubility after heating and improved retention of fluorescence or activity after heating to 100°C [31]. It should be noted that supercharging of the latter two proteins, while imparting thermal resilience, negatively impacted function. In further investigation of this method, the AvNAPSA approach for supercharging an scF_V_ did not lead to variants that could retain epitope binding after 70°C exposure for 1 hour.

One drawback of the automated AvNAPSA approach is that mutation of surface hydrophobic residues is disallowed, and decreasing the hydrophobic residue content is one route to alleviating aggregation [1]. Secondly, β-sheet propensity is another leading determinant of aggregation [32], and the automated AvNAPSA approach disallows mutation of I, V, T, F, and Y residues with high β-sheet propensity. Thirdly, solvent-exposed side chains sometimes form stabilizing contacts on the protein surface. For example, in the supercharged anti-MS2 scF_V_, the AvNAPSA protocol mutated a solvent-accessible native aspartate to arginine, though the aspartate side chain is predicted to form a hydrogen bond with a backbone amide in a surface loop [20] (**Figure S1**). A small percentage of surface-exposed residue mutations can still have significant deleterious effects on stability. In studies of ubiquitin, removal of charge-charge interactions ranged from having no effect to decreasing stability by several kcal/mol [33], [34]. Such variations, in addition to possible cooperative energetic effects [35], result in a weak correlation between accessible surface area and ΔΔG of folding [36]. Most experimental ΔΔG values for surface-exposed mutations fall between −1 and +2 kcal/mol [36]. These magnitudes are significant, especially upon heavy mutagenesis, compared to the marginal stability of most proteins. Thus, an automated strategy that removes surface interactions can work in some cases but not others.

Our approach to supercharging explicitly considers surface interactions when identifying acceptable residues for mutation. We employ the Rosetta computational modeling software [37], [38] to choose the residue positions and charged residue type to incorporate based on computed energies. The major terms of the full-atom energy function are Lennard-Jones attraction, Lennard-Jones repulsion, an implicit solvation model disfavoring burial of polar groups, hydrogen bonding, a statistical residue pair term for electrostatics, side-chain rotamer probability, and a reference energy used to favor native-like abundance of each amino acid type [39]. Thus, the Rosetta approach can preserve and potentially add stabilizing interactions on the surface while increasing net charge (**Figure S1**). In the Rosetta supercharging protocol, we use the score12 full-atom energy function [40] and manipulate the reference energies for arginine, lysine, aspartate, and glutamate to achieve varying levels of net charge (see Methods).

**Figure 1:**
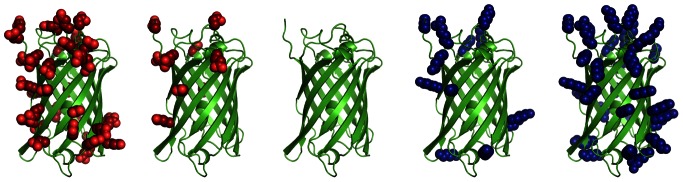
Illustration of supercharged GFP surfaces. The GFP backbone is shown in green cartoon, Asp/Glu side chains are shown in red spheres, Arg/Lys side chains are shown in blue spheres. **Left**: mutations in negatively-supercharged variants. **Center**: wild-type superfolding GFP. **Right**: mutations in positively-supercharged variants.

We ran Rosetta and AvNAPSA supercharging algorithms on 600 monomeric proteins from the Protein Data Bank and observed that the Rosetta protocol and AvNAPSA protocol give highly different designed sequences. To gauge the effectiveness of the Rosetta supercharge protocol, we characterized the expression, stability, and refolding of a series of GFP charge variants (**Figure 1**). Thermal denaturation of GFP results in irreversible aggregation, likely due to intermolecular β-sheet formation [41], [42]. Additionally, the absorbance and fluorescence signatures of the GFP chromophore provide a convenient way to monitor correct folding [43], [44], and GFP has been previously supercharged using the AvNAPSA solvent-accessibility approach [31]. Our results show that despite having highly different designed sequences, Rosetta supercharged GFP variants had similar expression, stability, and refolding properties as the AvNAPSA variants.

## Methods

### AvNAPSA Supercharge

**Figure 2:**
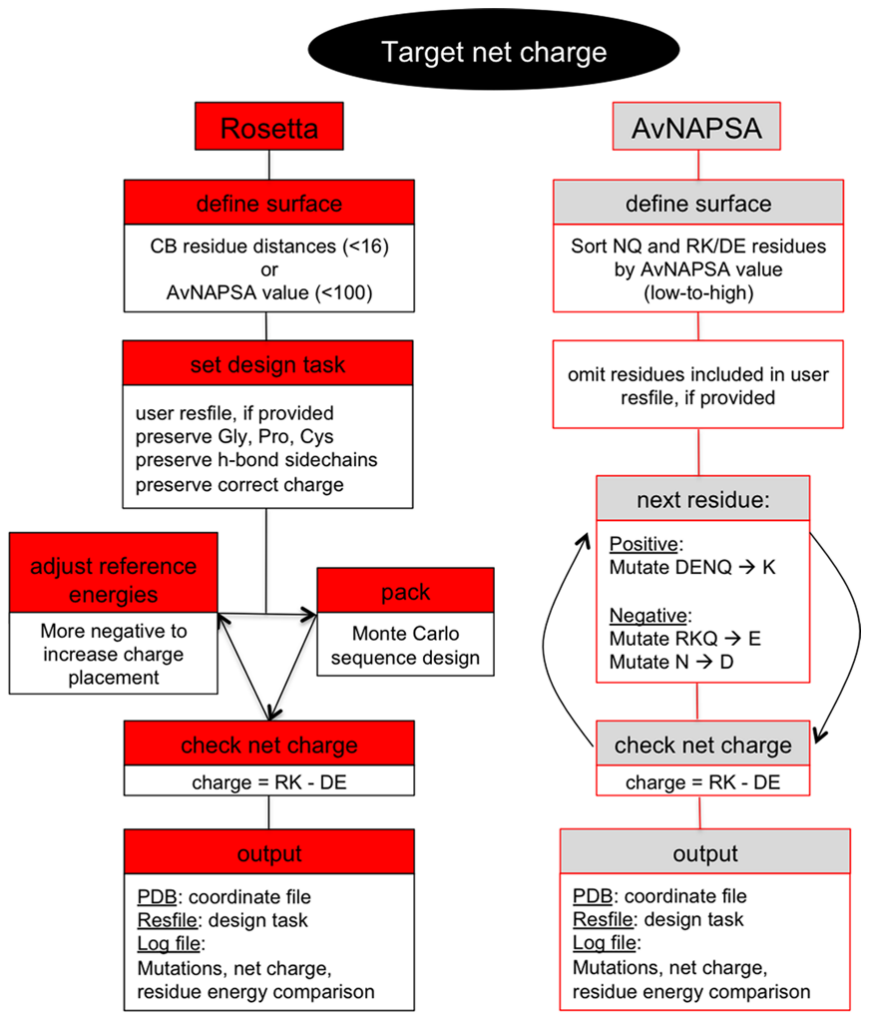
Workflow of two protocols for supercharging protein surfaces. Both protocols begin by defining the surface of the protein of interest, and if provided, reading a residue file that specifies residues to not mutate. AvNAPSA forcibly mutates NQ and DE/RK in order of solvent accessibility. Rosetta uses Monte Carlo side chain placement guided by computed energies to mutate any surface residue except G, P, C, and hydrogen-bonded side chains, and charged-residue reference energies are adjusted to vary net charge. Both protocols are set up to achieve a desired net charge (above), or to specified reference energies (Rosetta) or surface cutoff (AvNAPSA). Output includes the PDB coordinate file of the redesigned protein, the residue file specifying the allowed mutations, and a log file with information such as residues mutated, number of mutations, net charge, residue energies, and a PyMOL selection command to conveniently view the mutations.

Here we discuss two automated methods for supercharging, energy-based sampling with Rosetta and surface exposure rankings with AvNAPSA. The computational workflow in **Figure 2** illustrates the descriptions that follow. The previously demonstrated AvNAPSA supercharging protocol mutates the most highly solvent accessible NQ and DE/RK residues, where solvent accessibility is determined by the average neighbor atoms per side chain atom (AvNAPSA value) [31]. We implemented the AvNAPSA protocol within Rosetta, and to achieve a target net charge, the following workflow is used (**Figure 2**). First, all NQ and RK/DE residues are sorted by AvNAPSA value from low to high. One by one, the next residue in this sorted list is added to the list of mutations that will be made to the protein. If the user does not want specific residues to be mutated, this can be specified in an input file. Positive supercharging uses DENQ to K mutations, and negative supercharging uses RKQ to E and N to D mutations. Once the desired net charge is achieved, the Rosetta *PackRotamers* mover for sequence design uses the mutations list to generate the final sequence for output as a PDB coordinate file containing calculated residue energies. An alternative AvNAPSA mode is also available, where instead of specifying a target net charge, the user can specify a surface cutoff – AvNAPSA values <150 were used previously [31], AvNAPSA values <100 are appropriate for moderate supercharging.

### Rosetta Supercharge

Mutations of the most exposed residues will often impart minimal changes to the protein structure. However, by not considering energetic consequences of mutation, this approach may mutate surface residues involved in backbone or side-chain hydrogen bonds, negatively impacting overall stability. For example, D residues can interact with amide protons in turn/loop regions on the surface and N residues can cap either end of an alpha helix. Also, the AvNAPSA approach has been shown to miss opportunities to add stabilizing mutations by mutating partially buried residues (**Figure S1**). We propose an alternative strategy for supercharging surfaces that uses computed energy to choose mutations.

In the Rosetta approach, as with the AvNAPSA approach, the first step is to define the surface. This can either use the AvNAPSA surface definition, or the standard metric used in Rosetta – Rosetta typically defines surface residues as those having fewer than 16 neighboring residues with Cβ-Cβ distances <10 Å [45]. Using Cβ residue-based distances, the surface definition is insensitive to side-chain conformation or sequence changes. Surface definitions between the atom-based or residue-based neighbor calculations are noticeably different (R^2^ = 0.85, **Figure S2**), Rosetta supercharge uses the AvNAPSA surface definition by default. If the user wishes to not restrict mutations to the calculated surface – for example, a seemingly buried residue position could accommodate an arginine side chain that bends toward the surface – the surface definition can be increased to a residue neighbor cutoff of 30 or an AvNAPSA value of 200 to include peripheral or buried residues.

The next step of Rosetta supercharge is to set the design “task”, which specifies what amino acids are allowed or not allowed at each residue position. Residue positions included in a residue file, if provided by the user, will not be mutated (**Text S1**). This would be desirable if a known binding surface is important for function, or if a homology model is the only available starting structure. Starting from a homology model, mutating surface hydrophobic residues would be risky since these positions could actually be part of the core. Additional residues will also be preserved by default: those with the correct charge, those with side chains making a hydrogen bond (calculated hbond energy<−0.5 Rosetta energy units), and glycine, proline, and cysteine residues. The user can turn off any of these restrictions if desired.

The Rosetta supercharging protocol uses computed energies to choose surface mutations, and for this work, we use the score12 Rosetta energy function. Variations of the Rosetta energy function are used for special scenarios, such as DNA-protein interactions [46], consideration of hydrophobic patch size [45], low-resolution stages of protein folding [39], and incorporating constraints from experimental data [47]. However, for choosing surface mutations, we use the common-use energy function called score12. Although recent work has been done to optimize the Rosetta energy function [48], score12 has been the most consistently used and validated energy function for a variety of design goals.

The AvNAPSA approach varies net charge by adjusting the surface cutoff. In contrast, the Rosetta approach varies net charge by adjusting reference energies of the positive or negatively charged residue types when scoring protein sequences and conformations. The Rosetta energy function uses reference energies for all 20 amino acid types to provide residue bonuses/penalties that enable benchmark sequence recovery simulations to recapitulate residue frequencies in native proteins. In Rosetta supercharge, the reference energies for any of the included charged residue types can be specified, but the reference energies of the native residue types cannot be changed. The default weights in the score12 Rosetta energy function for R, K, D, and E are −0.98, −0.65, −0.67, and −0.81, respectively, but should be adjusted to give a spectrum of net charges (**Figure 1** **, Figure S3**). If desired, reference energies can be used to bias the choice between R v. K or D v. E. Alternatively, the user can specify a target net charge, and the protocol will iteratively increment the charged-residue reference energies until the desired net charge is achieved. Fixed backbone side chain placement of surface residues is often highly convergent, but the process is still stochastic so several runs can be performed using the ‘nstruct’ option. To summarize, the standard Monte Carlo *PackRotamers* mover and score12 Rosetta energy function govern the choice of mutations, but the reference energies can bias the choices to more or fewer charge mutations.

### ROSIE Supercharge Web Server with Rosetta and AvNAPSA Modes

**Figure 3:**
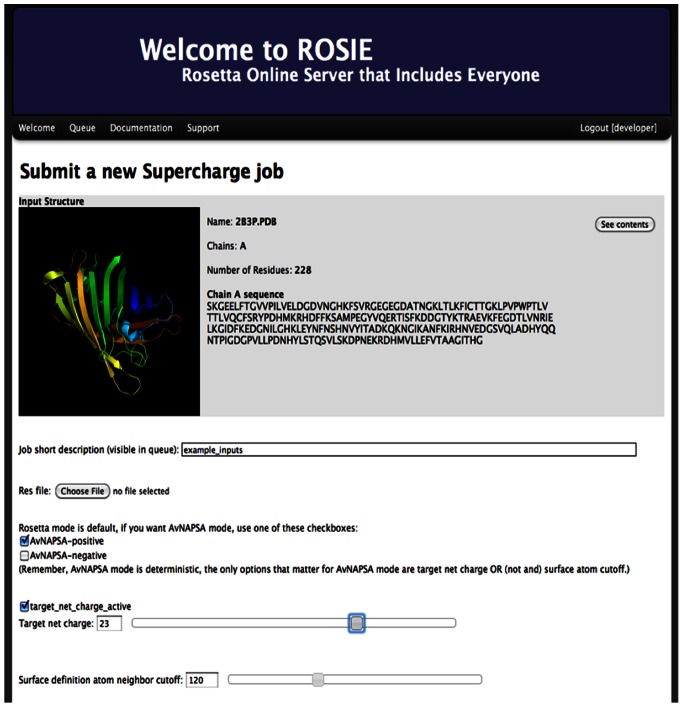
User interface for running the supercharge protocol on the ROSIE web server. The user uploads a PDB, then uses checkboxes or sliding bars to specify the protocol options, not all options are shown here (Table 1). Job status and protocol documentation can be viewed in the Queue and Documentation tabs.

Web servers have offered convenient and user-friendly access to Rosetta protocols [49], [50], [51]. The ROSIE web server (Rosetta Online Server Including Everyone) now provides a unifying framework for server implementation of Rosetta protocols [52]. To make both supercharging protocols broadly available, we implemented both protocols on the ROSIE web server (**Figure 3**). The AvNAPSA protocol can also be obtained as a perl script upon request from the Liu lab [53].

The supercharge protocol requires an input PDB in which all backbone atoms and a chain identifier should be present, and any unrecognized residues such as ligands will be ignored. The user can specify various options to use Rosetta or AvNAPSA mode, define the surface, choose a target net charge, and upload a residue file (resfile, Text S1); all options are listed in **Table 1**. We recommend that the user considers the starting net charge of the protein prior to supercharging: for input proteins starting with a negative net charge and low pI, negative supercharging will require fewer mutations to impart high net charge, and vice-versa. As output, a log file, the residue file that governed the design run, and the output PDB are provided. First, the log file contains the exact Rosetta command line, the residue positions identified as located on the surface, the number of each charged residue type in the final sequence, the net charge, a list of mutations, text for a PyMOL selection to easily view the mutations in PyMOL, and optionally, a full energetic comparison of repacked native versus supercharged structures. Secondly, the Rosetta residue file indicates which residue positions could possibly mutate, and to what residue types. The third output file is the atomic coordinate file of the supercharged protein, in PDB format, and the naming of the output PDB is intended to facilitate self-documentation of the inputs for a given design run. For Rosetta designs, the name includes the final reference energies and the final net charge, and for AvNAPSA designs, the name includes the net charge and the largest AvNAPSA value of the mutated residues.

**Table 1:** Rosetta supercharge options.

Either	option	default
bool	target_net_charge_active	false
int, signed	target_net_charge	0
int, unsigned	surface_atom_cutoff	120
file	resfile	N/A
bool	pre_packminpack	false
bool	compare_residue_energies_all	false
bool	compare_residue_energies_mut	true
**Rosetta**		
int, unsigned	surface_residue_cutoff	16
bool	include_arg	false
bool	include_lys	false
bool	include_asp	false
bool	include_glu	false
float	refweight_arg	−0.98
float	refweight_lys	−0.65
float	refweight_asp	−0.67
float	refweight_glu	−0.81
int, unsigned	nstruct	1
bool	preserve_glyprocys	true
bool	preserve_hbonded_sidechains	true
bool	preserve_correct_charge	true
**AvNAPSA**		
bool	AvNAPSA_positive	false
bool	AvNAPSA_negative	false

## Results

### Computed Energy Comparison between Rosetta and AvNAPSA Approaches

**Figure 4:**
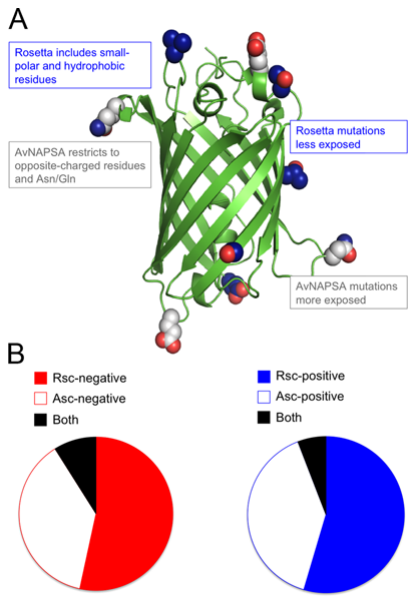
Rosetta and AvNAPSA supercharge protocols mutate different residues. **A)** AvNAPSA-mutated residue positions (white) are highly exposed and are often in loop regions, while Rosetta-mutated residue positions (blue) are less exposed and two mutations are in stable secondary structures. Native side chains of the mutated positions are shown in spheres to convey that Rosetta can mutate hydrophobic and small-polar residues. We emphasize that no mutations are shared between the two approaches in this low-charge design. **B)** Moderate supercharging was performed on 600 monomeric proteins, and the mutated residues were compared – each monomer was designed with the same net charge in both approaches. Rosetta requires more mutations to achieve the same net charge (solid v. empty). For negative-charge designs, 9% of mutated residue positions are shared (black, left). For positive-charge designs, 6% of mutated residue positions are shared (black, right). Shared mutations decrease an additional ∼2-fold considering that the chosen residue type differs ∼50% of the time for the shared residue positions – AvNAPSA never uses arginine, and AvNAPSA only uses aspartate if the native residue is asparagine.

The philosophy of the AvNAPSA supercharge approach is to minimize risk of perturbing the native structure while adding charged residues. The philosophy of the Rosetta supercharge approach is to maintain and possibly improve surface interactions while adding charged residues (**Figure 4A**). Using both approaches, large-scale positive- and negative-supercharging design runs on 600 proteins show how well each protocol accomplishes its goal, computationally. First, low-charge designs were generated with Rosetta using the default reference weights without specifying a target net charge; Rosetta could choose a charged residue or the native residue at each surface position. Then, for all 600 proteins, AvNAPSA was run to achieve the previous Rosetta net charges. Secondly, high-charge designs were generated with AvNAPSA using no target net charge and fixed surface cutoff (AvNAPSA value <150 as used previously [31]), then Rosetta was run to achieve the AvNAPSA-150 net charge for all 600 proteins. The low-charge variants averaged ∼7 mutations per structure, and the high-charge variants averaged ∼30 mutations per structure (**Figure S4, Table S1)**.

**Figure 5:**
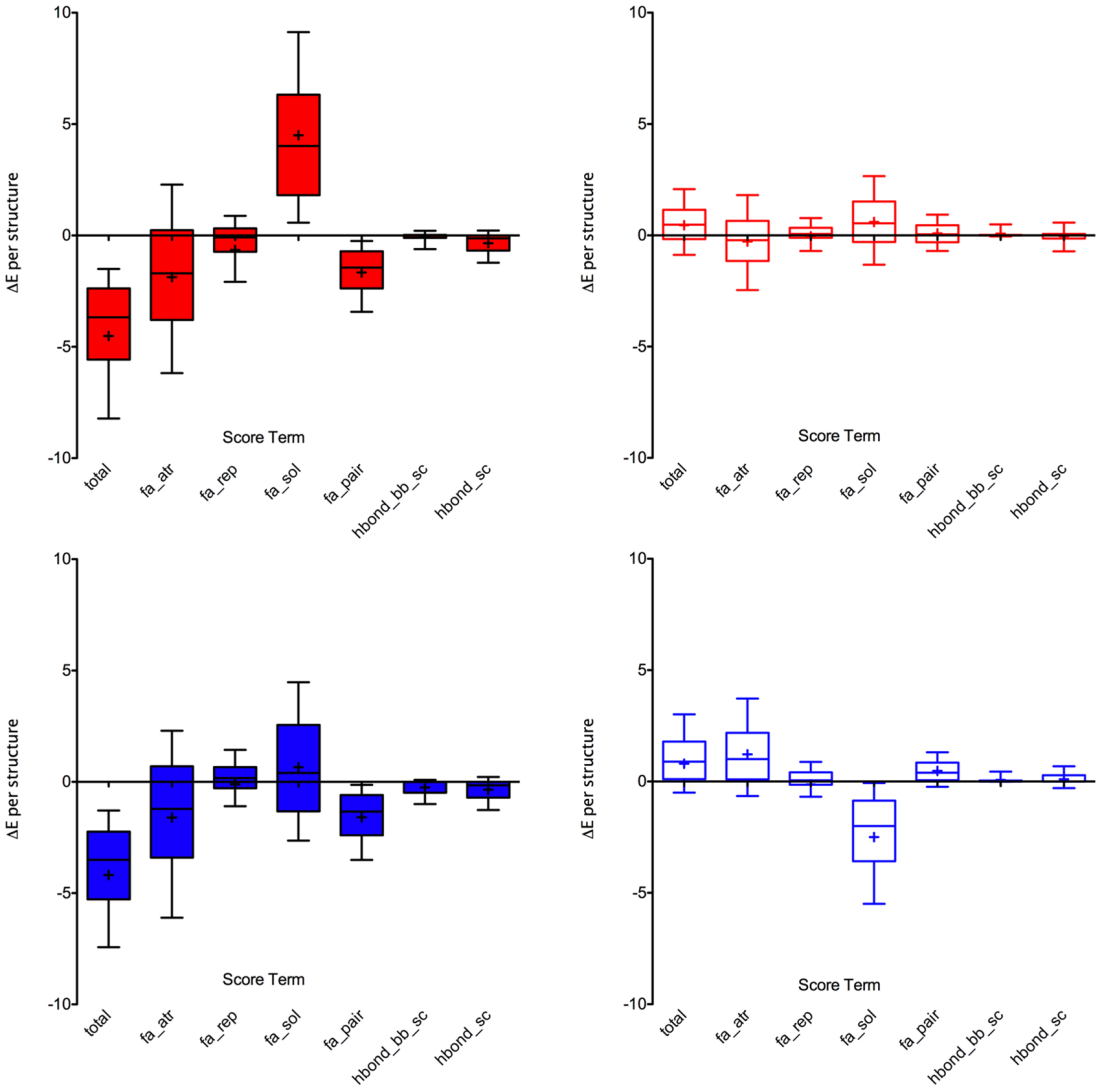
Low-charge variant residue energy changes per structure (600 total) broken down by each weighted score term. **Red**: negative-charge variants. **Blue**: positive-charge variants. **Solid bars**: Rosetta designs. **Empty bars**: AvNAPSA designs. AvNAPSA mutations have little effect on computed energy, on average (right, empty bars). Rosetta improves total energy primarily through Lennard-Jones attraction (fa_atr), charge complementarity (fa_pair), and reference energy, and a minor improvement results from addition of hydrogen bonds (left, solid bars). Rosetta mutations lead to increases in solvation energy (fa_sol) for negative supercharging. Not all score terms are included because their values cannot change in fixed backbone design (backbone-backbone hydrogen bonds, disulfides, proline closure, omega angle planarity). total: total residue energy, fa_atr: Lennard-Jones attraction, fa_rep: Lennard-Jones repulsion, fa_sol: Lazaridus-Karplus implicit solvation (penalizes buried polar atoms, slightly rewards buried carbon atoms), fa_pair: knowledge-based statistical term favoring oppositely-charged residues in close proximity, hbond_bb_sc: geometric score for backbone-sidechain hydrogen bonds, hbond_sc: geometric score for sidechain-sidechain hydrogen bonds.

**Figure 6:**
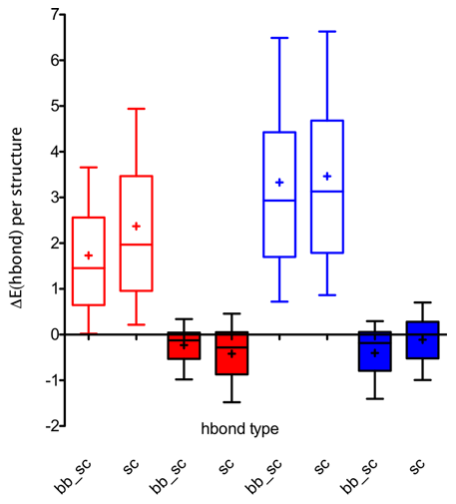
High-charge variant hydrogen bond energy changes per structure (600 total). In high-charge AvNAPSA designs (AvNAPSA cutoff of 150), removal of hydrogen bonds costs 1.5 to 3 Rosetta energy units per structure (empty bars). In contrast, Rosetta designs with the same net charge preserve hydrogen bonds (solid bars).

**Figure 7:**
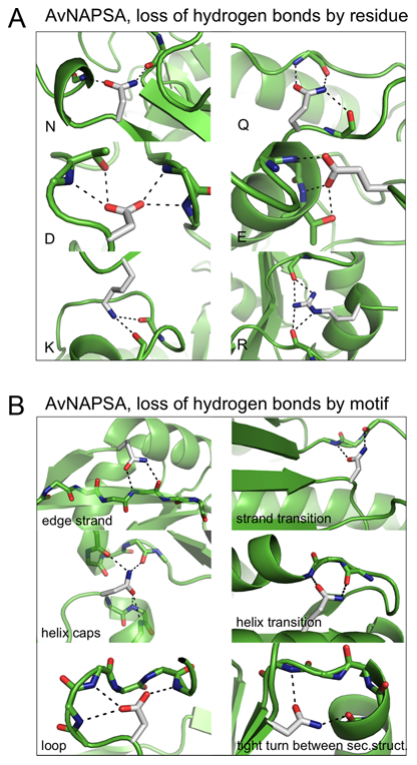
Specific examples of hydrogen bonds removed by AvNAPSA supercharge. In AvNAPSA designs, wild-type surface residues forming hydrogen bonds can be mutated (white sticks show the native side chain). **A)** Mutation of surface NQ/DE/RK residues can lead to loss of hydrogen bonds. **B)** Common sidechain-backbone hydrogen bonding motifs at protein surfaces mediate direct interaction with secondary structure elements and interaction with regions that transition between secondary structure elements. N and Q residues can act as both donor and acceptor, illustrating the risk of automated N to D and Q to E mutations.

In low-charge variants, the AvNAPSA approach on average has minimal effect on computed energies, except for an improved solvation energy for positive supercharging, which results from populating the highly exposed positions with lysines (**Figure 5**). In high-charge variants, however, the AvNAPSA approach removes attractive interactions, adds repulsive interactions, and places like-charges in close proximity (**Figure S4**). Also, several surface hydrogen bonds per structure are lost (**Figure 6** **,** **Figure 7**). The specific examples in **Figure 7** are for illustrative purposes; on average, high-charge AvNAPSA designs removed 3 strong hydrogen bonds and 8 weak hydrogen bonds per structure. High-charge Rosetta designs added 0.25 strong hydrogen bonds and 1.6 weak hydrogen bonds per structure (**Table S1**). As expected, the Rosetta approach improves the Rosetta scores because it chose mutations based on these computed scores (**Figure 5**). The Lennard-Jones attractive term and the knowledge-based pair term show improvements – the pair term favors placing oppositely-charged residues near each other. Hydrogen bonding improves only slightly (**Figure 5** **, Figure S5, Table S1**).

These changes in computed energy are informative but expected. The striking comparison between these two approaches is the extent of dissimilarity between chosen mutations. For low-charge supercharging, the two approaches only share 6–9% of mutated residue positions (**Figure 4B**). Furthermore, the shared mutations decrease by about half when only counting residue positions that were mutated to the same residue type. Why do these two surface redesign protocols diverge to such a great extent? In positive supercharging, Rosetta can mutate 15 amino acid types: DE-NQ-ASTHMVLIYFW, while AvNAPSA can mutate 4 amino acid types: DE-NQ (**Figure S6**). This effectively allows AvNAPSA to build a higher charge with a lower mutational load (**Figure S8**), but it allows Rosetta more choices for energetically favorable mutations. Secondly, among the DE-NQ residues that both protocols are allowed to mutate, Rosetta is inclined to mutate partially buried positions (**Figure S7**) that can add additional van der Waals contacts, charge complementarity, or hydrogen bonds, while AvNAPSA attempts to “leave-not-a-trace”, to have minimal effect on protein surface contacts (**Figure 4**). Thirdly, the fully automated AvNAPSA protocol only uses K while Rosetta uses K and R for design.

### Expression and Foldedness of Supercharged GFP Variants

We observed that the Rosetta and AvNAPSA protocols for supercharging lead to highly dissimilar designed sequences. We then experimentally characterized a series of positive- and negatively-supercharged variants of GFP from the Rosetta approach (RscG). Here we demonstrate that a highly dissimilar computed energy-based method can also lead to improved refolding, but we add caution that severe mutagenesis and/or charge (>33 mutations, higher than +40 or −43 in this study), even when limited to the surface, is likely to impair expression and proper folding. We note that the previously described AvNAPSA GFP variants (AscG−30 and AscG+36) were not actually designed using the fully-automated AvNAPSA approach described above, though AvNAPSA values were the primary input for choosing residue mutations. Visual inspection was also used, and AscG−30 was derived from a library screen that mixed wild-type and AscG−39 oligonucleotides because AscG−39 did not express [31]. Thus, the experimental results are not rigorous comparisons between methods, but the AscG−30 and AscG+36 variants offer a metric of success for evaluation of Rosetta variants.

**Figure 8:**
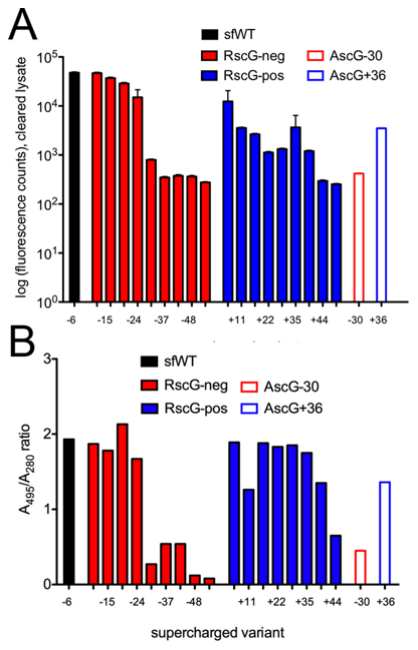
Expression and relative folding of supercharged GFP variants. **A)** Total fluorescence values indicate the level of expression of correctly folded GFP. The low- to mid-charge negative variants expressed well, but mid- to high-charge variants expressed significantly worse that sfWT. **B)** Absorbance ratios indicate the relative amount of correctly folded GFP. Absorbance by the chromophore at 495 nm indicates correctly folded GFP, and absorbance at 280 nm indicates the total amount of GFP. Low- to mid-charge variants are well folded (before thermal challenge), while high-charge variants are not well folded.

We tested RscG variants ranging from −11 to −61 and +7 to +58 with the number of mutations ranging from 6 to 49 (**Table 2**). For reference, the starting net charge of wild-type superfolder GFP (sfWT) is −6 [54]. Detailed methods of GFP construct assembly and expression are given in Text S1. Auto-induced bacterial cultures were grown (24 hours, 37°C) and normalized according to absorbance at 600 nm. Following sonication and centrifugation, each cleared lysate was scanned at emission/excitation wavelengths of 488/509 nm to gauge the level of expressed, correctly folded soluble GFP. Wild-type sfGFP and negative variants extending to charges of −24 expressed comparably well (**Figure 8A**). Expression levels dropped precipitously beyond this net charge (variants RscG−32 to RscG−61, as well as AscG−30). Moderate expression was observed with positively charged variants ranging from +7 through +40, while the RscG+44 and RscG+58 designs expressed poorly. Again, the AvNAPSA variant AscG+36 exhibited expression similar to its Rosetta counterpart, RscG+35. These experiments were performed in physiological salt concentrations of 150 mM. Resolubilization of the insoluble pellet in 5 M NaCl recovered a large fraction of properly folded, fluorescent protein, particularly in the higher net positive charge range (**Figure S9**). As a second measurement of correct folding, the GFP variants were purified and ratios of absorbance at 488 nm (folded GFP) versus absorbance at 280 nm (total protein) were determined. Most Rosetta supercharged variants had similar A_488_/A_280_ ratios as sfWT except for the high-charge negative variants RscG−32 to −61 and the highest-charge positive variant RscG+58 (**Figure 8B**).

**Table 2:** Mutations and net charge of supercharged GFP variants.

Protein	Reference energy 1	Reference energy 2	Net charge	# mutations	Δ computed energy**
sfWT			−6	0	0
RscG−11	−0.27	−0.41	−11	6	−3.5
RscG−15	−0.42	−0.56	−15	9	−4.2
RscG−18	−0.52	−0.66	−18	11	−5.2
RscG−24	−0.67	−0.81	−24	15	−5.7
RscG−32	−0.82	−0.96	−32	20	−4.6
RscG−37	−0.97	−1.11	−37	23	−3.5
RscG−43	−1.22	−1.36	−43	28	−1.8
RscG−48	−1.42	−1.56	−48	33	+0.9
RscG−52	−1.67	−1.81	−52	36	+3.5
RscG−61	−1.67*	−1.81*	−61	49	+6.2
RscG+7	−0.74	−0.41	+7	9	−5.3
RscG+11	−0.94	−0.61	+11	12	−4.6
RscG+15	−1.09	−0.76	+15	15	−4.6
RscG+22	−1.14	−0.81	+22	19	−4.0
RscG+27	−1.19	−0.86	+27	22	−3.6
RscG+31	−1.24	−0.91	+31	25	−1.9
RscG+35	−1.34	−1.01	+35	28	−0.6
RscG+40	−1.54	−1.21	+40	32	+0.8
RscG+44	−1.79	−1.46	+44	35	+2.9
RscG+58	−1.99*	−1.66*	+58	47	+6.0
AscG−30			−30	15	+4.5
AscG+36			+36	29	+6.9

* hydrogen bonded side chains allowed to mutate.

** with default reference energies.

### Stability and Refolding of Supercharged GFP Variants

**Figure 9:**
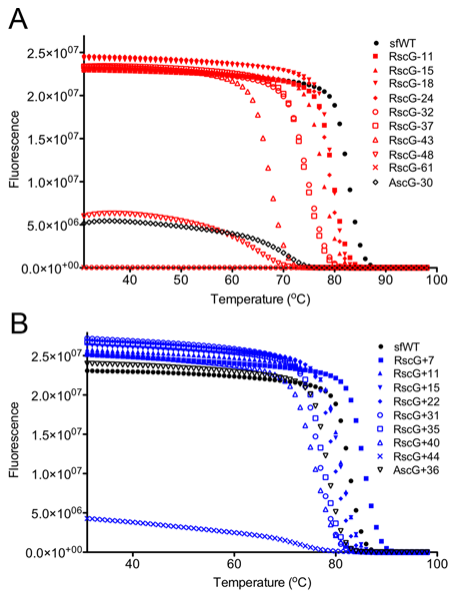
Stability measurements using thermal denaturation while monitoring GFP fluorescence. **A)** negative-charge variants. **B)** positive-charge variants. Rosetta-based designs retain thermostability within 10°C of sfWT, except for the variants requiring severe mutagenesis (>33 mutations).

Following purification by immobilized metal ion affinity chromatography, supercharged variant concentrations were normalized to 2 µM by A_280_. GFP fluorescence was monitored during thermal denaturation to assess the impact of Rosetta supercharging on stability. Moderately charged variants up to RscG−24 and RscG+31 exhibited melting transitions within 5°C of wild type. RscG negatively-charged variants were more stable than the AscG−30 variant, which supports the use of computed energies to choose mutations. Beyond RscG−32 and RscG−35, the more highly negative charges of −43 and −48 showed significantly impaired stability (**Figure 9A**). In contrast, the positively supercharged variants were more robust, a charge of +44 was reached before severe destabilization occurred (**Figure 9B**).

**Figure 10:**
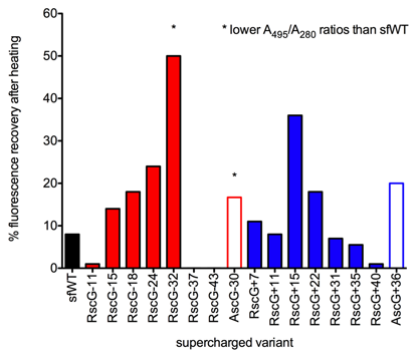
Percent recovery of fluorescence after heating GFP variants to 95°C for 3 minutes. All variants were tested at 2 µM concentration. Some variants demonstrated poor A_495_/A_280_ ratios and should not be directly compared to sfWT (RscG−32 and AscG−30). Improvements to refolding are 3-fold for RscG−24 and 4.5-fold for RscG+15.

Additional experiments were performed to assess refolding after thermal denaturation. 2 µM samples of the GFP variants were measured for initial fluorescence, then heated to 95°C for 1 to 5 minutes, then monitored for fluorescence recovery at room temperature over the course of 20 minutes. The length of incubation at 95°C significantly impacted recovered fluorescence – for wild-type, 60% recovery occurred after 1 minute of heating, compared to 8% after 3 minutes and <5% after 5 minutes of heating (**Figure S10**). Similar trends were observed for supercharged variants, though the effect of incubation time was not as pronounced. Recovered fluorescence increased for negatively supercharged variants up to RscG−32, after which RscG−37 and RscG−43 appeared not to refold at all (**Figure 10**). RscG−32 exhibited a 50% recovery in fluorescence, similar to the 39% recovery of AscG−30 (**Figure S11**). For positively supercharged variants, charges up to +40 were well tolerated and did not negatively impact refolding. However, only two variants, RscG+15 and RscG+22 improved fluorescence recovery to 40% and 20%, respectively. RscG+35 exhibited 6% recovery, and AscG+36 exhibited 20% recovery (**Figure 10**).

## Discussion

Supercharging protein surfaces should aid a variety of applications, such as improving thermoresistance and refolding yield [16], [20], [31], and in the case of positively supercharged proteins, enabling cellular entry [27], [53]. Supercharging is challenging because the deleterious effects of successive mutations eventually overcome the plasticity of a surface and hamper protein function. In contrast to an approach based on surface exposure only, the Rosetta supercharge protocol uses computed energy to introduce mutations, potentially avoiding decrements in stability and leading to more functional supercharged proteins.

We have previously used Rosetta to both positively and negatively supercharge antibodies [20] and wished to further show the generality of this method. In this regard, green fluorescent protein was an especially attractive target for engineering due to its common use, fluorescence readout, and poor refolding. In addition, we sought to better understand determinants of charge-dependent refolding by comparing our Rosetta energy-based approaches with the AvNAPSA residue-exposure supercharging method that had previously been applied to GFP [31]. Although certain Rosetta variants showed slightly better thermostability and fluorescence recovery than AvNAPSA variants, marginal differences in a study of limited scope cannot substantiate claims that one method outperforms the other. The goal of this study was to propose an alternative approach to a previously demonstrated approach.

Both supercharging methods have their advantages and disadvantages. The AvNAPSA approach requires fewer mutations to achieve the target net charge due to higher likelihood of a charge swap – AvNAPSA only requires 0.6 mutations per charge while Rosetta requires 0.85 mutations per charge, on average (**Figure S8**). However, the Rosetta approach can mutate exposed hydrophobic residues to charged polar residues, and removing surface hydrophobic residues can help prevent aggregation of partially unfolded states. As a caveat, the expanded choice of positions to mutate may lead to the inadvertent discovery of destabilizing mutations, especially with wild-type residues that are partially buried. AvNAPSA mutations can also be destabilizing, due to loss of sidechain-sidechain and sidechain-backbone hydrogen bonds when mutating exposed residues. Several common surface sidechain-backbone hydrogen bonding motifs are important for structure and stability: 1) direct interaction with secondary structure elements: edge-strand interaction, helix capping, loop stabilization; and 2) interaction with transitions between secondary structure elements: stand entry/exit, helix entry/exit, tight turns between secondary structures (**Figure 7**). Furthermore, N and Q residues can serve simultaneously as donors and acceptors, and in these cases mutation to D and E are destabilizing (**Figure 7**). Lastly, Rosetta can choose between arginine and lysine and preserve/add stabilizing interactions unique to arginine [55], [56], [57], while the automated AvNAPSA approach uses only lysine.

Although native surface hydrogen bonds are safeguarded by computed energies, surface interactions remain challenging to accurately model and score. Likely magnified in supercharged designs, one major gap in the current Rosetta scoring function is the lack of a physics-based term to describe long range [58]electrostatic interactions. Electrostatic calculations that solve the Poisson-Boltzmann equation are computationally expensive and cannot be evaluated using rapid pair-wise scoring. Instead, Rosetta uses a knowledge-based pair term that disfavors placing like-charged residues and favors placing oppositely-charged residues in close proximity. This knowledge-based pair term crudely captures cation-pi interactions between arginine and aromatic residues [59], but there is currently not a cation-pi term in the Rosetta score function.

Thus, there are two different strategies for supercharging a surface: partially capture surface energetics (Rosetta), or ignore error-prone energy calculations and attempt to minimize the mutagenic footprint (AvNAPSA). The Rosetta and AvNAPSA protocols (Rsc and Asc) diverged when choosing surface mutations, but both protocols led to GFP variants with improved refolding. Many RscG variants retained native-like stability, while the AscG−30 variant was destabilized. In general, variants with intermediate net charges (20–30 net charge for a 28 kDa protein) tended to refold better than low- or high-charge variants. However, we were not able to pinpoint more precise reasons that some designs worked better than others. For example, variant RscG−32 demonstrated the best refolding, while variant RscG−37 did not refold. Our protocols were not uniformly successful because consequences of mutations are challenging to predict. Even when only mutating two residues, energy changes upon removing or adding charge-charge interaction on protein surfaces can vary highly depending on the protein [1], [33], [34], [60], [61], and on location on the protein surface [34]. Furthermore, the risk of mutating a critical surface residue increases with more mutations. The number of mutations can be limited by adding like-charges according to the starting net charge or pI rather than reversing the charge sign of the input protein. In our study, 20+ mutations decreased expression yield and stability. Consistent with these observations, the initial negatively supercharged GFP variant designed by AvNAPSA, AscG−39, contained 20 mutations, but did not express well in E. *coli*.

Because of these uncertainties in surface energy calculations, optimal net charge, and consequences of mutating many residues, another possible approach to improve refolding of a target protein is to augment computational design with directed evolution or high-throughput screening. In fact, the AscG−30 variant was generated by a randomization and screening approach. Since the negatively supercharged AscG−39 variant did not express in *E. coli*, it was shuffled with wild-type GFP to generate a library. This library was screened by picking fluorescent colonies for sequencing, and the most fluorescent variant had 15 of the original 20 mutations [31], [53].

In summary, we have developed a Rosetta-based protocol for supercharging protein surfaces. GFP variants with intermediate net charges (20–30 net charge for a 28 kDa protein) tended to refold better than low- or high-charge variants. We conclude that computational methods to find the best sequence for refolding are partially successful, and for future uses of supercharging to improve refolding, we recommend testing a series of variants or combining computational design with high-throughput screening to identify successful variants.

## Supporting Information

Figure S1 **Motivation for considering surface interactions when choosing charge mutations**. Above are computational models of scFv supercharge designs. **Left**: By only considering solvent accessibility, surface hydrogen bonds may be lost. In a positive-supercharge design, the AvNAPSA method removed an aspartate that was making a sidechain-backbone hydrogen bond in a surface loop. **Right**: In a positive-supercharge design, Rosetta mutated a partially buried residue to add a salt-bridge hydrogen bond.
(TIF)

Figure S2 **Atom-based versus residue-based definition of surface residues**. Rosetta typically defines surface residues as having <16 neighboring residues with Cβ- Cβ distances <10 Å. The AvNAPSA protocol is named after how it defines surface residues: by the Average Neighboring Atoms Per Sidechain Atom (10 Å neighbor distance cutoff). The residue-based definition is not sensitive to change in sequence or sidechain rotamer. These two definitions can vary in which residues are identified as part of surface, and the Rosetta-supercharge protocol can use either definition. Values in the plot are derived from surface definitions of 600 monomeric proteins.
(TIF)

Figure S3 **Top**: Rosetta supercharge varies net charge by adjusting the reference energy of the desired charged-residue types. **Bottom**: AvNAPSA varies net charge by adjusting the atom-based surface cutoff (AvNAPSA value). GFP is represented in green cartoon, and arginine/lysine mutations are represented in blue spheres. Wedges represent increasing/decreasing net charge.
(TIF)

Figure S4 **Computed energy changes for high-charge variants for each score term**. AvNAPSA variants had a fixed surface cutoff (AvNAPSA value <150), and Rosetta variants were designed to reach the same net charge. AvNAPSA variant energies get worse in many terms (empty bars), while Rosetta variant energies are preserved (solid bars). Rosetta variants were designsed using altered reference energies but were scored using the default reference energies. See Figure 5 of the main text for the same analysis of low-charge variants.
(TIF)

Figure S5 **Rosetta can place charged side chains to form new hydrogen bonds**. Relevant side-chain and backbone atoms are shown in sticks, Rosetta mutations are colored orange, wild-type side chains and backbones are colored green, and hydrogen bonds are represented in black dashes.
(TIF)

Figure S6 **Rosetta can mutate 15 residue types, AvNAPSA can mutate 4 residue types**. AvNAPSA conservatively mutates exposed flexible polar residues for minimal change to the surface characteristics (empty bars). When searching for favorable mutations, Rosetta can mutate all residue types except glycine, proline, and cysteine (solid bars). Mutating surface hydrophobic residues, for example, reduces hydrophobic content and might help prevent aggregation of the unfolded state.
(TIF)

Figure S7 **Residues mutated by Rosetta supercharge have more atom neighbors than residues mutated by AvNAPSA supercharge**. AvNAPSA, by definition, targets residues with lowest AvNAPSA values. Rosetta mutates less-exposed residues to add more favorable contacts.
(TIF)

Figure S8 **AvNAPSA requires fewer mutations to accomplish a target net charge**. AvNAPSA mutations are limited to NQ and DE/RK residues, giving a ∼50% chance of a charge swap. Rosetta can mutate many uncharged residues, so it requires closer to one mutation per charge addition.
(TIF)

Figure S9 **Recovery of fluorescent GFP from the pellet after centrifugation of cell lysates**. After lysis and centrifugation, treatment of pelleted fractions with 5 M NaCl increased yields of positively-charged GFP variants.
(TIF)

Figure S10 **Superfolder GFP (sfGFP) refolding is diminished by increased incubation times at 95°C**. High-temperature incubation at 1 minute leads to >50% refolding, while incubation at 5 minutes leads to <5% refolding.
(TIF)

Figure S11 **Percentage of fluorescence recovered while recovering at 25°C after heating to 95°C for 3 minutes**. Rosetta variants (bold lines) and AvNAPSA variants (thin lines) show similar refolding percentages. The negative variants Asc-30 and Rsc-32 have lower A495/A280 ratios than sfWT, so percent refolding is not a fair metric to compare these designed variants and sfWT. sfGFP refolds to 8% fluorescence recovery.
(TIF)

Table S1 **Number of computed hydrogen bonds lost/gained per supercharged structure.**
(DOC)

Text S1 **Supporting information.**
(DOC)
