# Supplementary figures and images for: Alternative Computational Protocols for Supercharging Protein Surfaces for Reversible Unfolding and Retention of Stability
Source: PLoS One. 2013 May 31;8(5):e64363. doi: 10.1371/journal.pone.0064363 (PMC3669367; doi:10.1371/journal.pone.0064363)

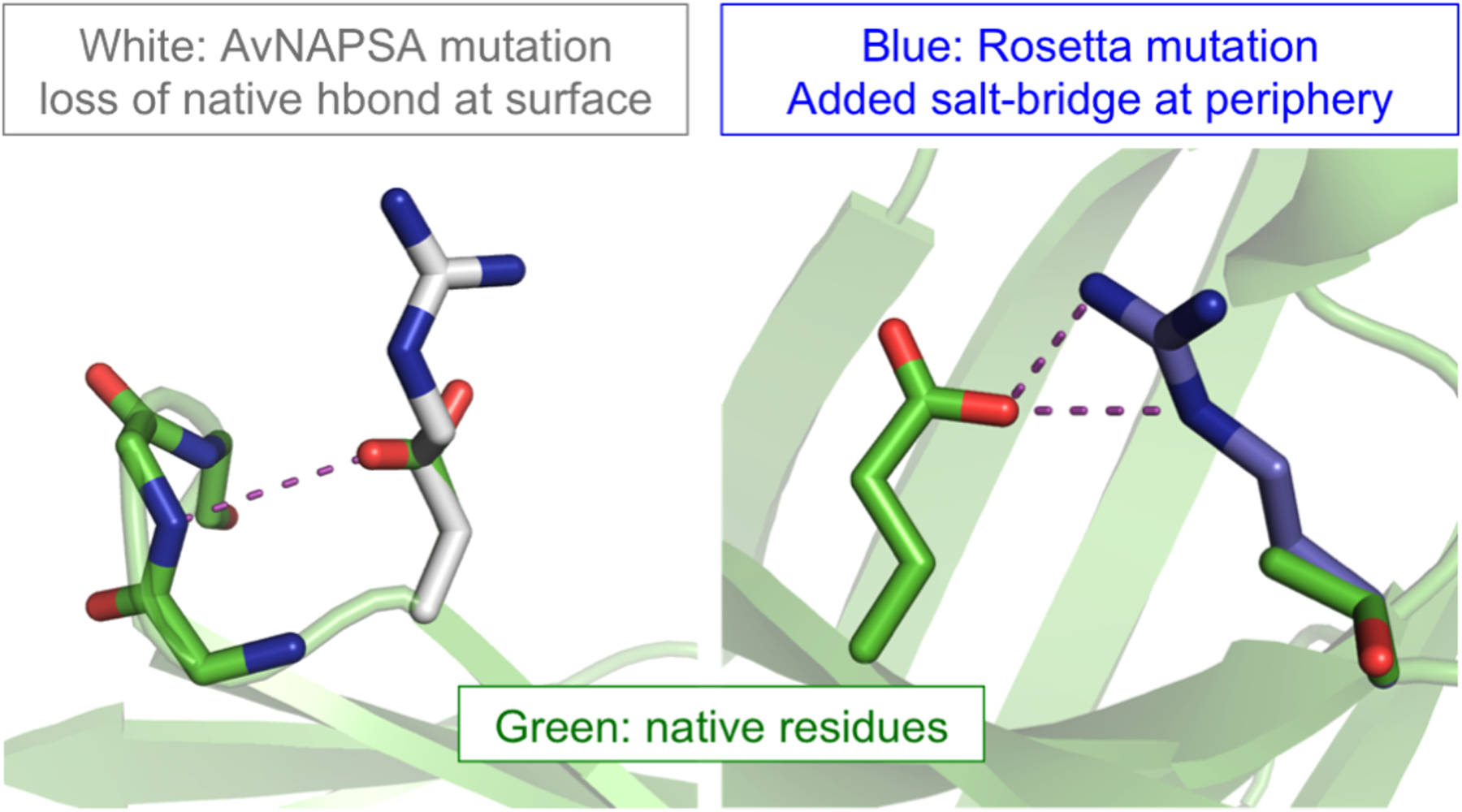

Supplement: Figure S1 — Motivation for considering surface interactions when choosing charge mutations. Above are computational models of scFv supercharge designs. Left: By only considering solvent accessibility, surface hydrogen bonds may be lost. In a positive-supercharge design, the AvNAPSA method removed an aspartate that was making a sidechain-backbone hydrogen bond in a surface loop. Right: In a positive-supercharge design, Rosetta mutated a partially buried residue to add a salt-bridge hydrogen bond. (TIF) [file pone.0064363.s001.tif]

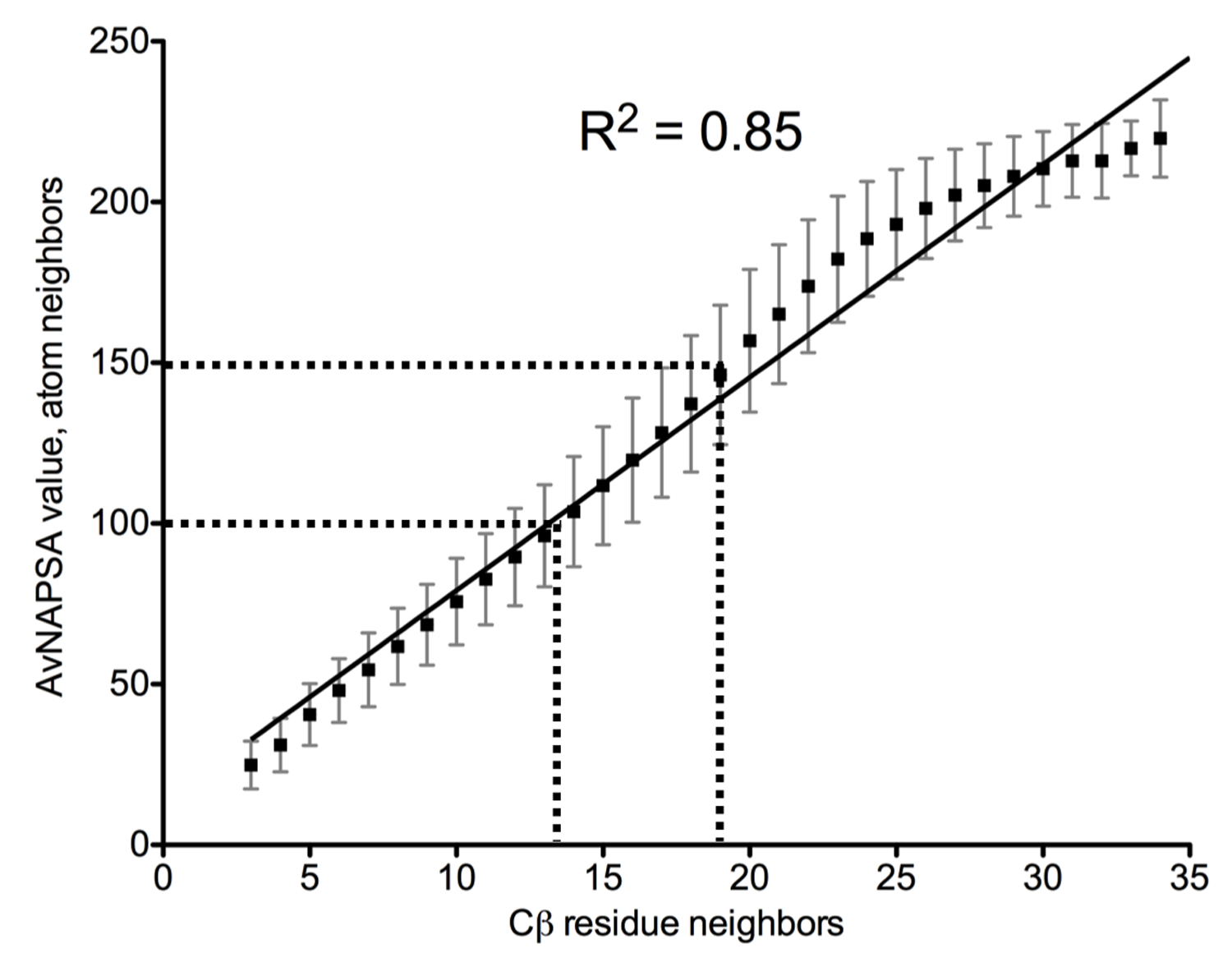

Supplement: Figure S2 — Atom-based versus residue-based definition of surface residues. Rosetta typically defines surface residues as having <16 neighboring residues with Cβ- Cβ distances <10 Å. The AvNAPSA protocol is named after how it defines surface residues: by the Average Neighboring Atoms Per Sidechain Atom (10 Å neighbor distance cutoff). The residue-based definition is not sensitive to change in sequence or sidechain rotamer. These two definitions can vary in which residues are identified as part of surface, and the Rosetta-supercharge protocol can use either definition. Values in the plot are derived from surface definitions of 600 monomeric proteins. (TIF) [file pone.0064363.s002.tif]

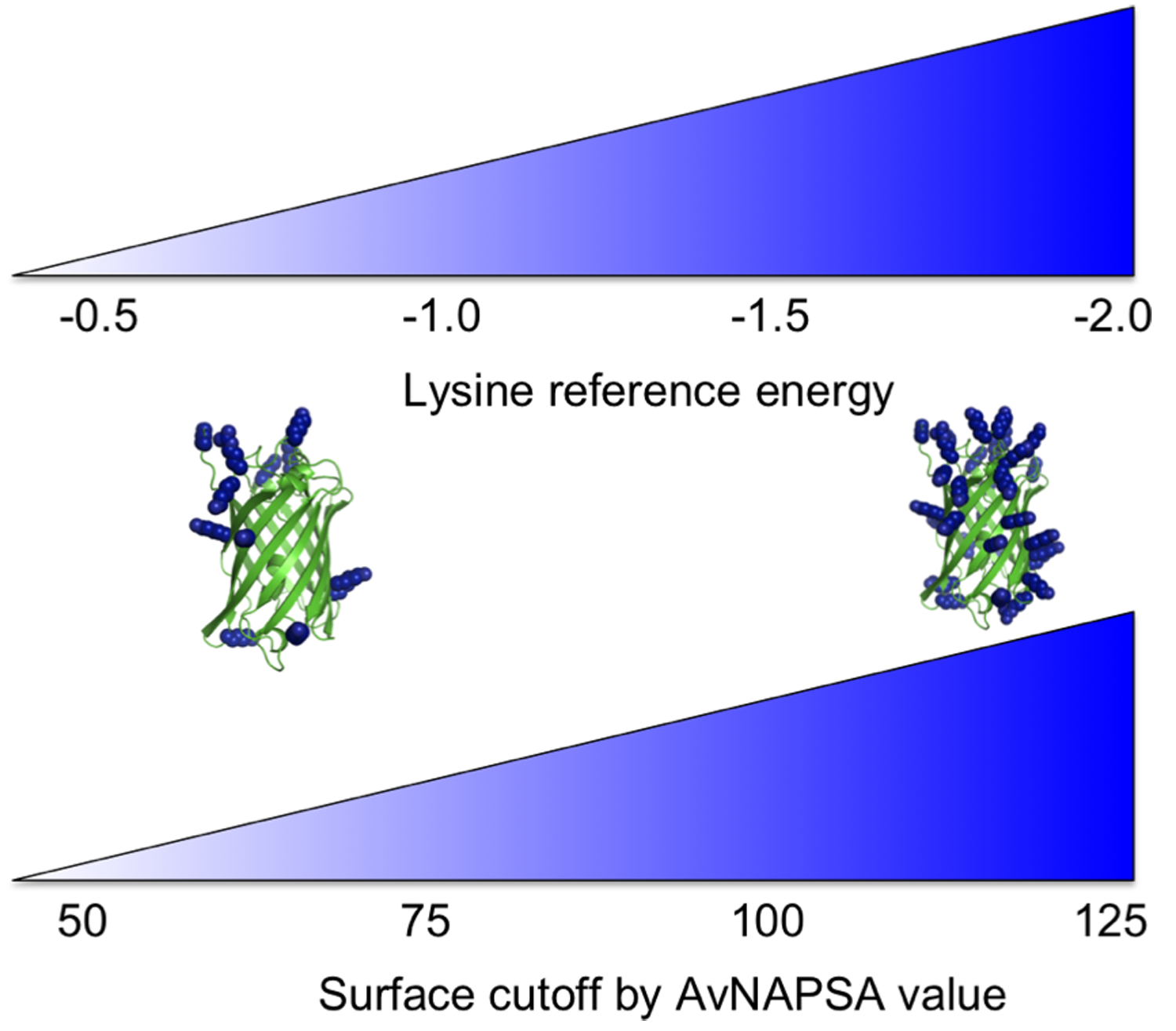

Supplement: Figure S3 — Top: Rosetta supercharge varies net charge by adjusting the reference energy of the desired charged-residue types. Bottom: AvNAPSA varies net charge by adjusting the atom-based surface cutoff (AvNAPSA value). GFP is represented in green cartoon, and arginine/lysine mutations are represented in blue spheres. Wedges represent increasing/decreasing net charge. (TIF) [file pone.0064363.s003.tif]

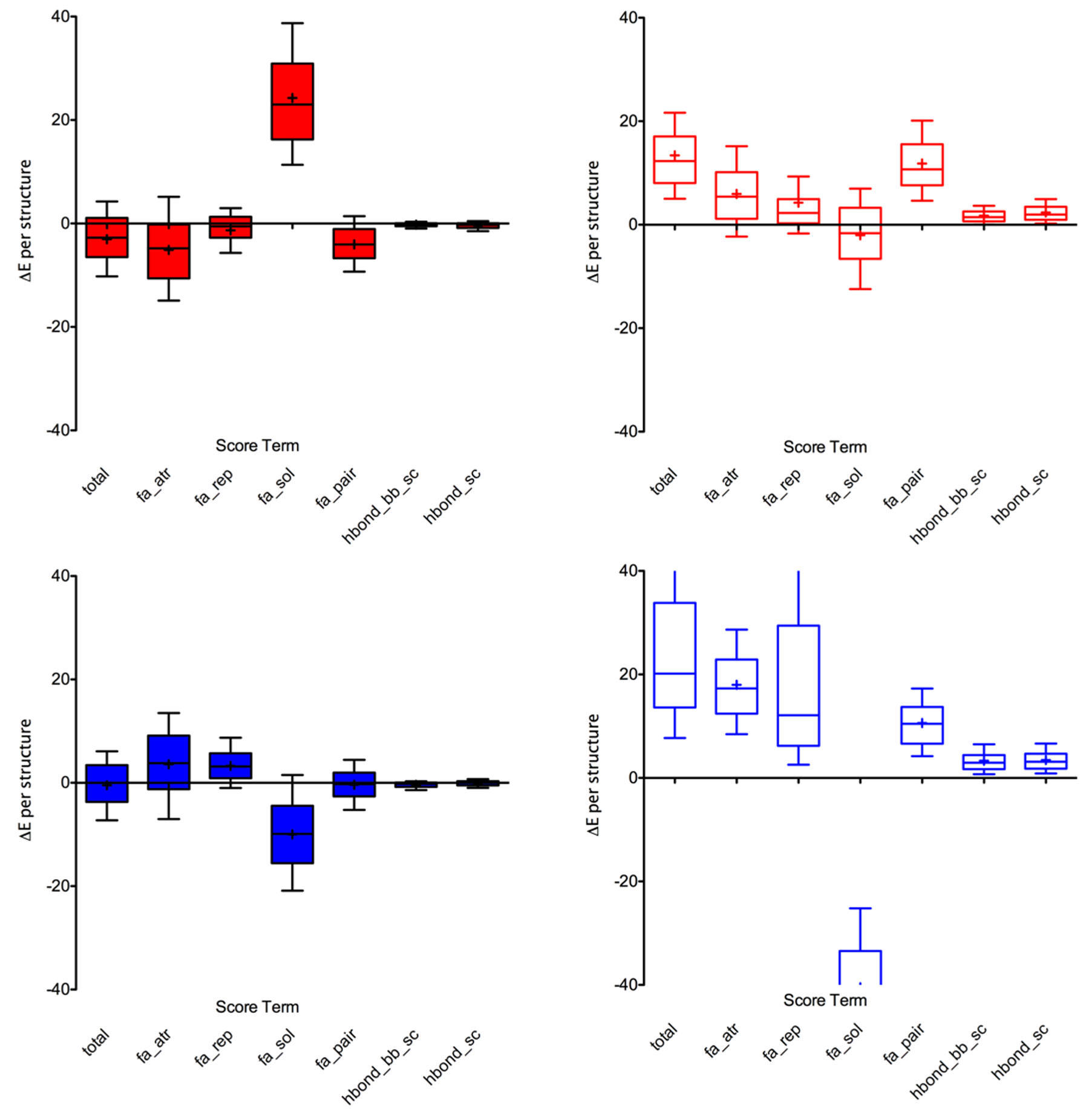

Supplement: Figure S4 — Computed energy changes for high-charge variants for each score term. AvNAPSA variants had a fixed surface cutoff (AvNAPSA value <150), and Rosetta variants were designed to reach the same net charge. AvNAPSA variant energies get worse in many terms (empty bars), while Rosetta variant energies are preserved (solid bars). Rosetta variants were designsed using altered reference energies but were scored using the default reference energies. See Figure 5 of the main text for the same analysis of low-charge variants. (TIF) [file pone.0064363.s004.tif]

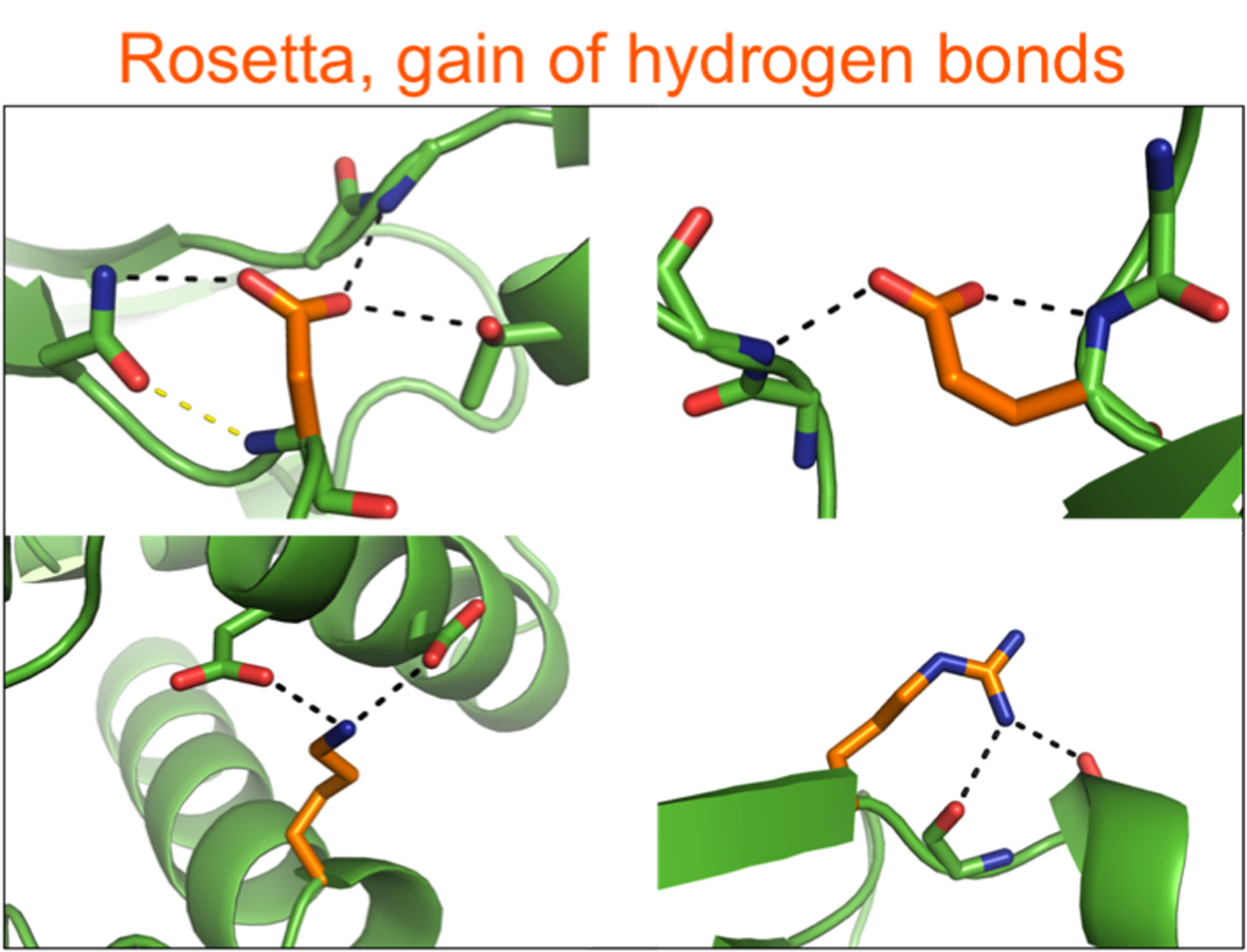

Supplement: Figure S5 — Rosetta can place charged side chains to form new hydrogen bonds. Relevant side-chain and backbone atoms are shown in sticks, Rosetta mutations are colored orange, wild-type side chains and backbones are colored green, and hydrogen bonds are represented in black dashes. (TIF) [file pone.0064363.s005.tif]

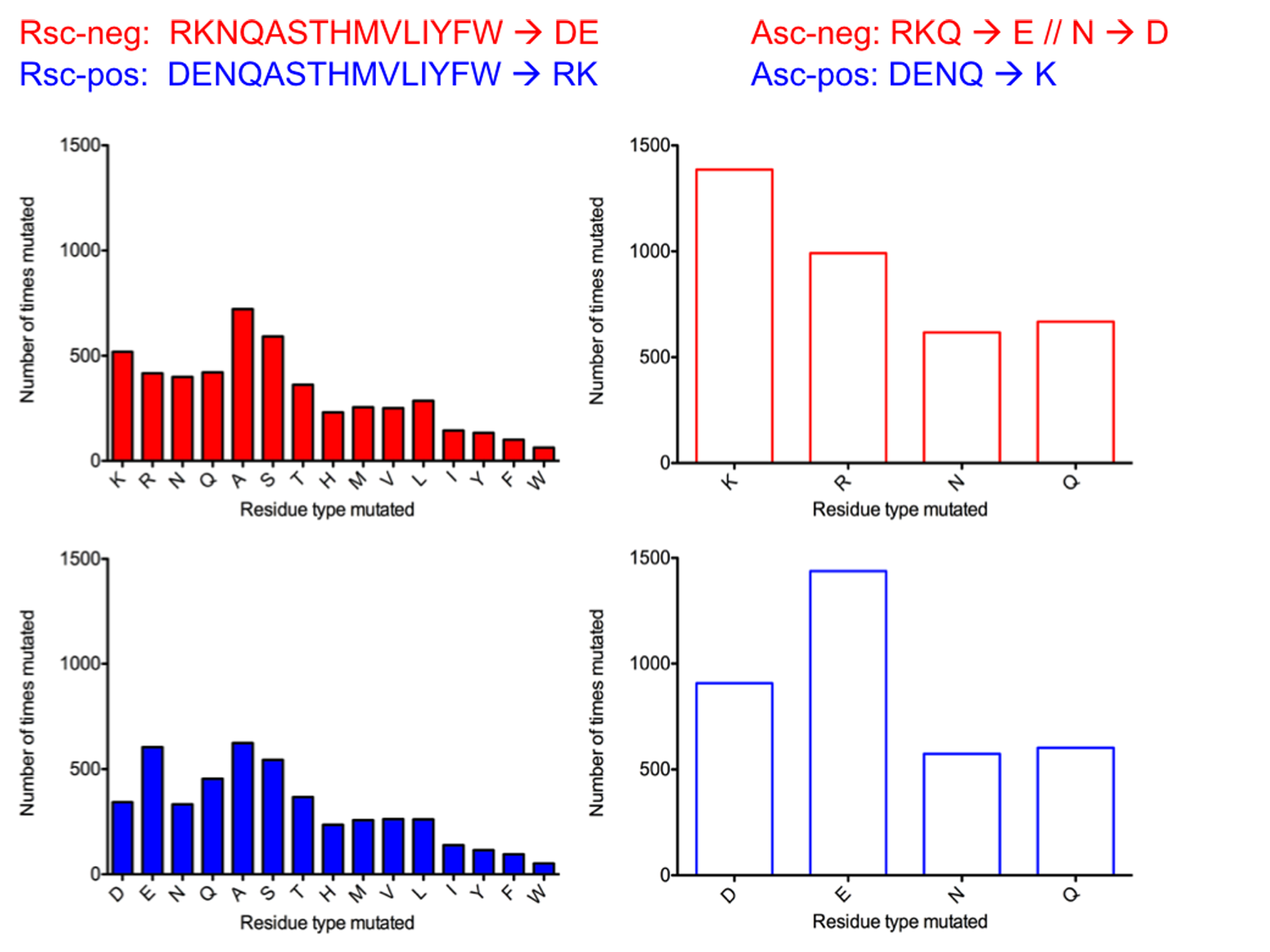

Supplement: Figure S6 — Rosetta can mutate 15 residue types, AvNAPSA can mutate 4 residue types. AvNAPSA conservatively mutates exposed flexible polar residues for minimal change to the surface characteristics (empty bars). When searching for favorable mutations, Rosetta can mutate all residue types except glycine, proline, and cysteine (solid bars). Mutating surface hydrophobic residues, for example, reduces hydrophobic content and might help prevent aggregation of the unfolded state. (TIF) [file pone.0064363.s006.tif]

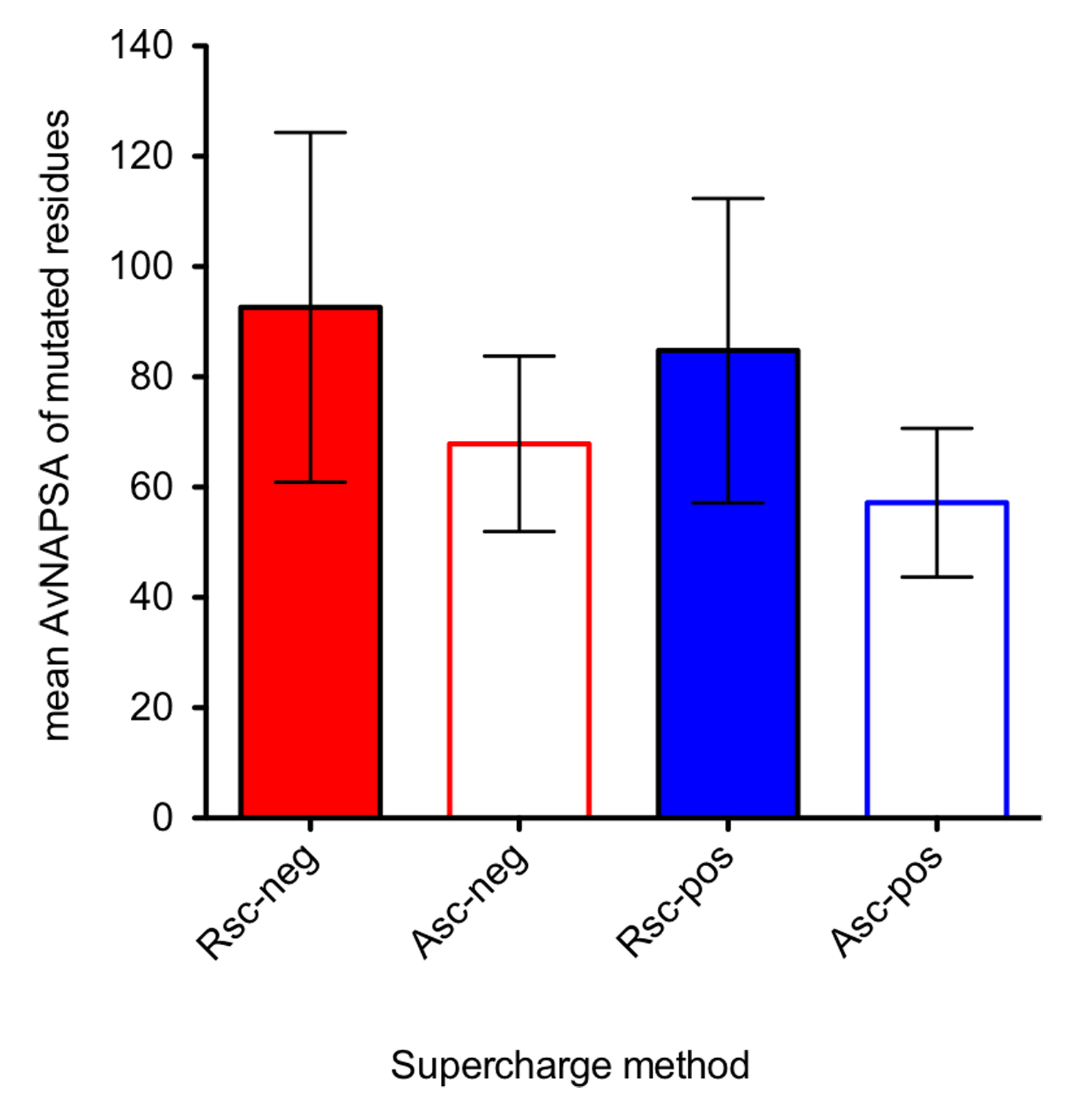

Supplement: Figure S7 — Residues mutated by Rosetta supercharge have more atom neighbors than residues mutated by AvNAPSA supercharge. AvNAPSA, by definition, targets residues with lowest AvNAPSA values. Rosetta mutates less-exposed residues to add more favorable contacts. (TIF) [file pone.0064363.s007.tif]

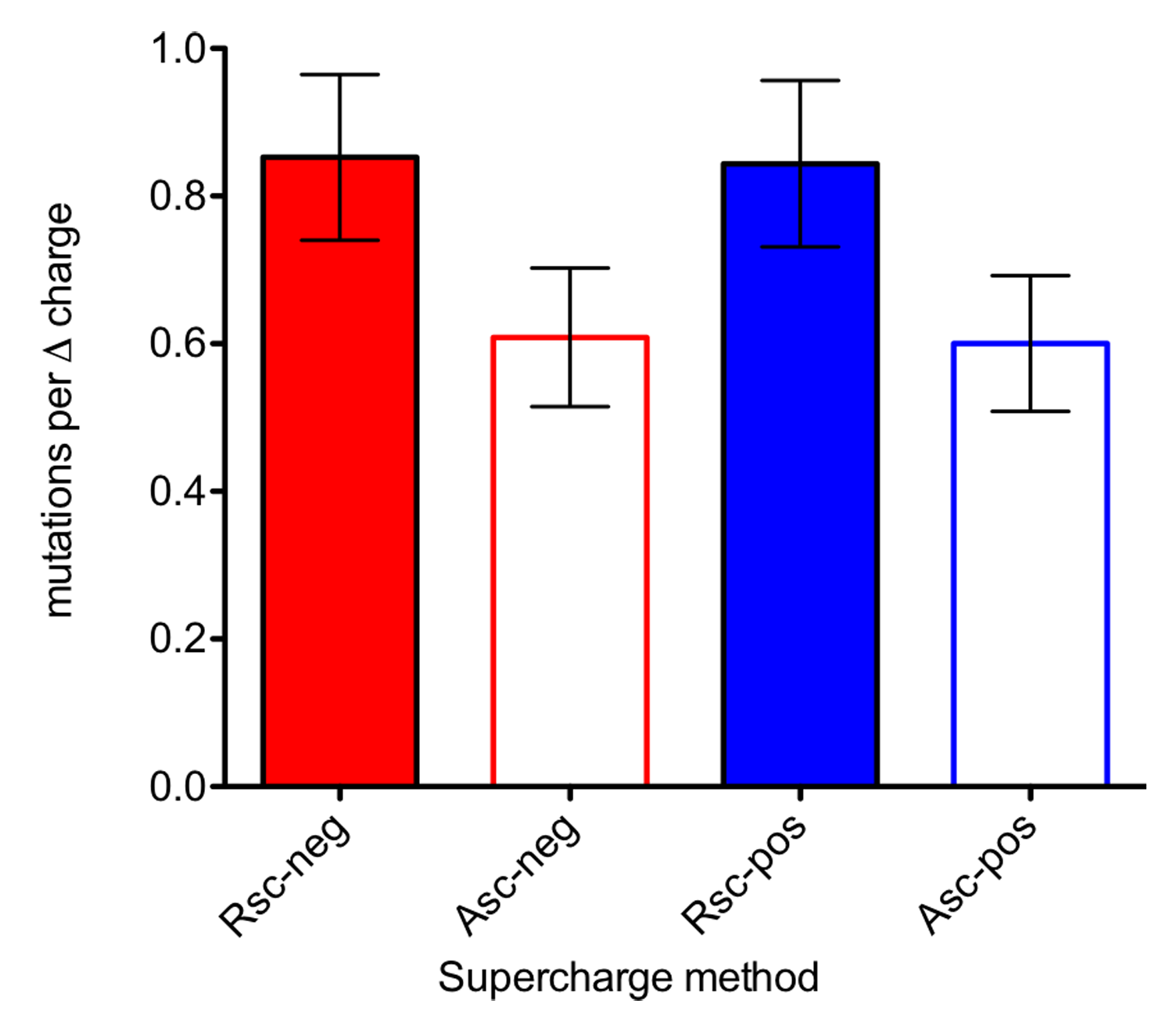

Supplement: Figure S8 — AvNAPSA requires fewer mutations to accomplish a target net charge. AvNAPSA mutations are limited to NQ and DE/RK residues, giving a ∼50% chance of a charge swap. Rosetta can mutate many uncharged residues, so it requires closer to one mutation per charge addition. (TIF) [file pone.0064363.s008.tif]

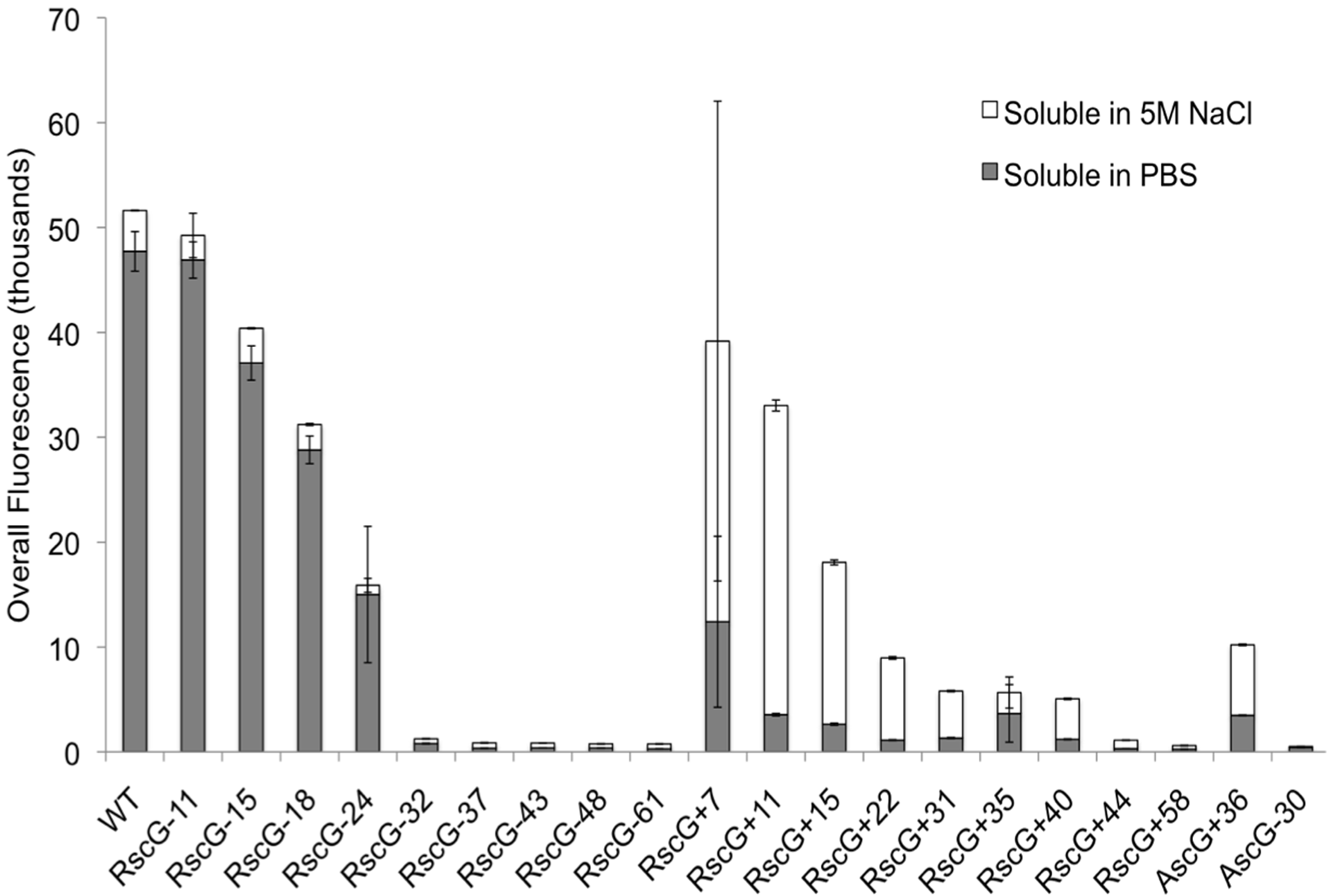

Supplement: Figure S9 — Recovery of fluorescent GFP from the pellet after centrifugation of cell lysates. After lysis and centrifugation, treatment of pelleted fractions with 5 M NaCl increased yields of positively-charged GFP variants. (TIF) [file pone.0064363.s009.tif]

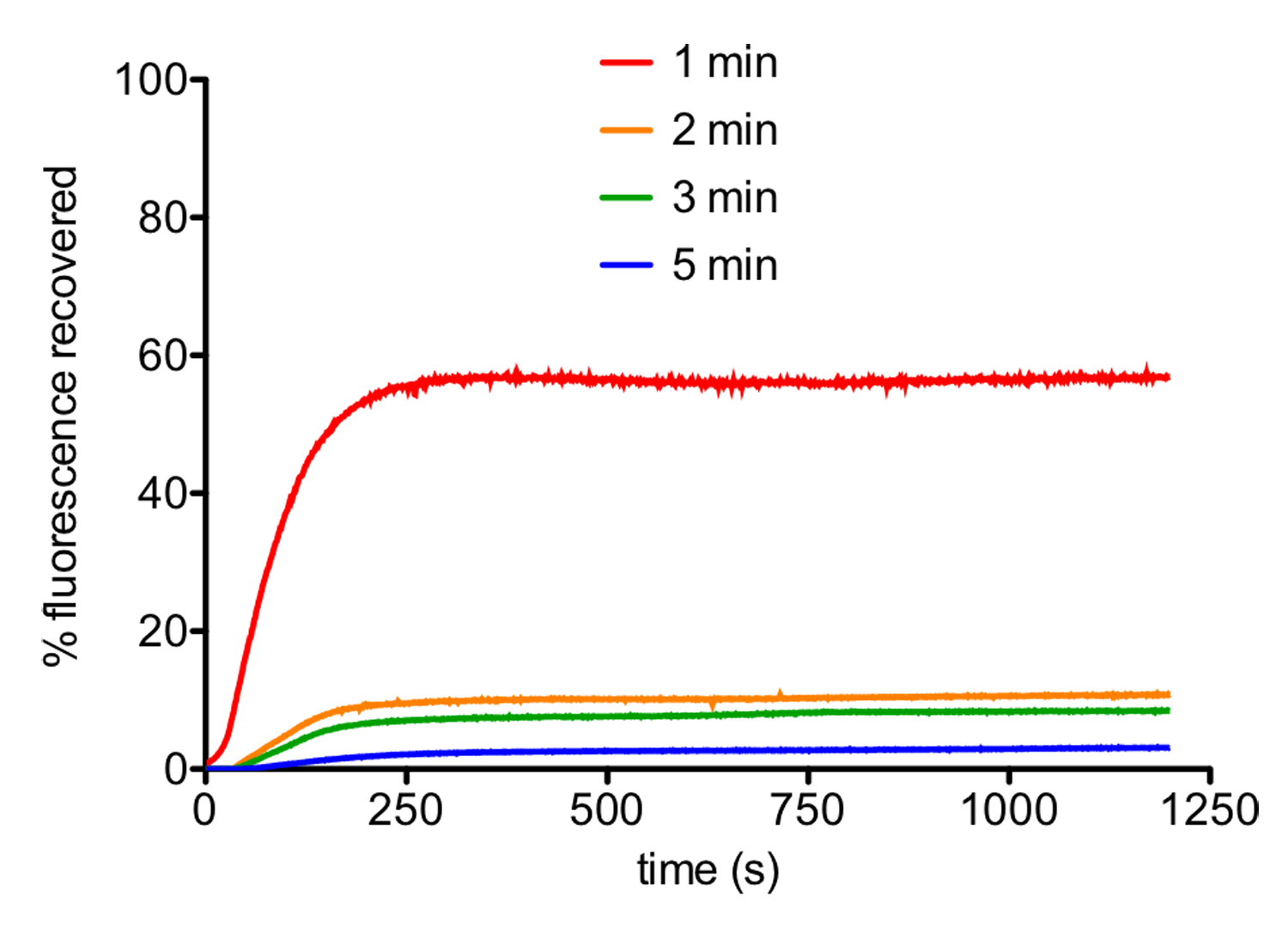

Supplement: Figure S10 — Superfolder GFP (sfGFP) refolding is diminished by increased incubation times at 95°C. High-temperature incubation at 1 minute leads to >50% refolding, while incubation at 5 minutes leads to <5% refolding. (TIF) [file pone.0064363.s010.tif]

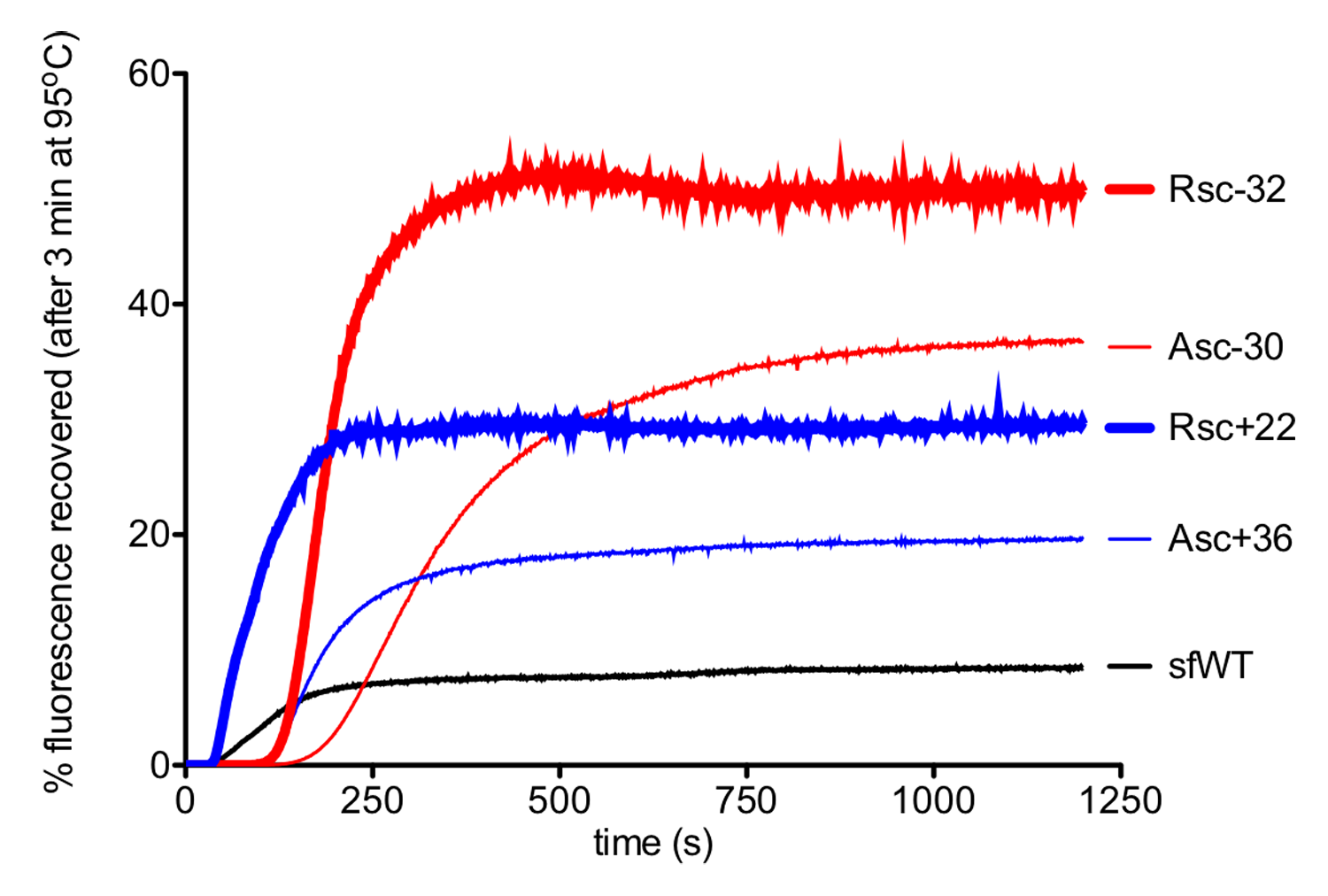

Supplement: Figure S11 — Percentage of fluorescence recovered while recovering at 25°C after heating to 95°C for 3 minutes. Rosetta variants (bold lines) and AvNAPSA variants (thin lines) show similar refolding percentages. The negative variants Asc-30 and Rsc-32 have lower A495/A280 ratios than sfWT, so percent refolding is not a fair metric to compare these designed variants and sfWT. sfGFP refolds to 8% fluorescence recovery. (TIF) [file pone.0064363.s011.tif]
