# Supplementary material for: Alternative Computational Protocols for Supercharging Protein Surfaces for Reversible Unfolding and Retention of Stability
Source: PLoS One. 2013 May 31;8(5):e64363. doi: 10.1371/journal.pone.0064363 (PMC3669367; doi:10.1371/journal.pone.0064363)
Supplement: Table S1 — Number of computed hydrogen bonds lost/gained per supercharged structure. (DOC) [file pone.0064363.s012.doc]

**Table S1**. Number of computed hydrogen bonds lost/gained per supercharged structure

| **lost** | Asc-neg | Asc-pos | **gained** | Rsc-neg | Rsc-pos |
| --- | --- | --- | --- | --- | --- |
| strong hbond* | -2.3 | -3.5 | strong hbond | +0.21 | +0.26 |
| weak hbond** | -5.9 | -9.9 | weak hbond | +1.6 | +1.7 |

*strong hbond: ΔE_hbond < -0.5 Rosetta energy units

**weak hbond: -0.25 > ΔE_hbond > -0.5 Rosetta energy units
