# Supplementary material for: Alternative Computational Protocols for Supercharging Protein Surfaces for Reversible Unfolding and Retention of Stability
Source: PLoS One. 2013 May 31;8(5):e64363. doi: 10.1371/journal.pone.0064363 (PMC3669367; doi:10.1371/journal.pone.0064363)
Supplement: Text S1 — Supporting information. (DOC) [file pone.0064363.s013.doc]

Supporting Information

for

Alternative computational protocols for supercharging protein surfaces for reversible unfolding and retention of stability

Bryan S. Der,^1^ Christien Kluwe,^2,3^ Aleksandr E. Miklos,^2,3,4^ Ron Jacak,^1^ Sergey Lyskov,^5^ Jeffrey J. Gray,^5^ George Georgiou,^3^ Andrew D. Ellington,^2,3,4^ Brian Kuhlman,^1,6,‡^

^1^ Department of Biochemistry and Biophysics, University of North Carolina at Chapel Hill, Chapel Hill, North Carolina, United States of America

^2^ Center for Systems and Synthetic Biology, University of Texas at Austin, Austin, Texas United States of America

^3^ Institute for Cellular and Molecular Biology, University of Texas at Austin, Austin Texas, United States of America

^4^ Applied Research Laboratories, University of Texas at Austin, Austin, Texas, United States of America

^5^ Department of Chemical and Biomolecular Engineering, Johns Hopkins University, Baltimore, Maryland, United States of America

^6^ Lineberger Comprehensive Cancer Center, University of North Carolina at Chapel Hill, Chapel Hill, North Carolina, United States of America

^‡^ Corresponding author

Brian Kuhlman, Ph.D.
Phone: (919) 843-0188
Fax: (919) 966-2852
[bkuhlman@email.unc.edu](mailto:bkuhlman@email.unc.edu)

**Instructions for running the supercharge protocol in Rosetta:**

1. **Preprocessing of input files**

There is minimal preprocessing to run the supercharge protocol.

--relaxing the PDB, if you want

The supercharge protocol always pre-packs the surface before design. However, the user has the option of pack-minimization-pack. This will do a more extensive optimization of the input structure, but it is not necessary. If desired, the user can use other Rosetta protocols such as ‘relax’ to optimize the input structure before running supercharge.

--Removing ligands/waters from your input PDB is optional.

Ligands and waters will be ignored by the flag ‘ignore_unrecognized_res’. If you have ligands in your input structure, be wary of mutations in that region. In most cases, ligands are safely ignored because supercharge by default is usually run without moving the backbone. The ‘pre_packminpack’ option allows only one step of backbone minimization.

1. **Command line examples / Running the application**

Test runs can be performed on Rosetta-readable PDB files using these example options files:

The full list of command line options is given in Table 1 of the main text.

The options can be used to specify the following runs:

1. Rosetta-mode, no target charge (reference weights determine charge)
2. Rosetta-mode, target charge
3. AvNAPSA-mode, no target charge (surface_atom_cutoff determines charge)

AvNAPSA-mode calculates an AvNAPSA value for every residue. ‘surface_atom_cutoff’ indicates the cutoff AvNAPSA value that defines surface residues. AvNAPSA values of 50-150 are typical for surface residues. AvNAPSA values >150 are typical for core residues. A surface_atom_cutoff of 100 will lead to moderate supercharging. A surface_atom_cutoff of 150 will lead to heavier supercharging.

1. AvNAPSA-mode, target charge

Options file, example 1:

#positive supercharging, Rosetta-mode

#surface definition by residue neighbors

#no target net charge, net charge determined by refweights

-database <path_to_database>

-s your_pdb_file.pdb

-use_input_sc

-jd2:no_output

-ignore_unrecognized_res

-surface_residue_cutoff 16

-include_arg

-include_lys

-refweight_arg -1.98

-refweight_lys -1.65

-dont_mutate_glyprocys true

-dont_mutate_correct_charge true

-dont_mutate_hbonded_sidechains true

-nstruct 2

-compare_energies

-only_compare_mutated_residues

-resfile rosetta_inputs/resfile

-out:path example_outputs/outpath1

Options file, example 2:

#negative supercharging, Rosetta-mode

#surface definition by atom neighbors

#target net charge

-database <path_to_database>

-s your_pdb_file.pdb

-use_input_sc

-jd2:no_output

-ignore_unrecognized_res

-surface_atom_cutoff 100

-pre_packminpack true

-include_asp

-include_glu

-refweight_asp -0.6

-refweight_glu -0.8

-target_net_charge_active

-target_net_charge -20

-dont_mutate_glyprocys true

-dont_mutate_correct_charge true

-dont_mutate_hbonded_sidechains true

-out:path example_outputs/outpath2

Options file, example 3:

#negative supercharging, AvNAPSA-mode

#target net charge

-database <path_to_database>

-s your_pdb_file.pdb

-use_input_sc

-jd2:no_output

-ignore_unrecognized_res

-AvNAPSA_negative

-target_net_charge_active

-target_net_charge -25

-out:path example_outputs/outpath3

Options file, example 4:

#positive supercharging, AvNAPSA-mode

#no target net charge, net charge determined by surface cutoff

-database <path_to_database>

-s your_pdb_file.pdb

-use_input_sc

-jd2:no_output

-ignore_unrecognized_res

-AvNAPSA_positive

-surface atom_cutoff 120

-out:path example_outputs/outpath4

Example input residue file (note: an output resfile also governs the design task).

Line 1: default of the input resfile should be ALLAA. Note: The protocol generates an output resfile with NATAA as the default setting. This sets all core positions to NATAA. This output file combined with the optional input resfile govern the design run.

Line 2: start

Column 1: residue number

Column 2: chain identifier

Column 3: setting

ALLAA: all amino acids

NATAA: native amino acid

NATRO: native rotamer (fixed sidechain)

ALLAA

start

20 A NATAA

24 A NATAA

28 A NATAA

30 A NATRO #will preserve native hbond

1. **Postprocessing**

PDB file of supercharged protein:

The PDB file containing the coordinates of the redesigned protein can be loaded in pymol along with the input PDB to visually inspect the mutations.

Log file:

What residues are part of the surface:

supercharge: adding 5 to surface set

supercharge: adding 8 to surface set

supercharge: adding 9 to surface set

supercharge: adding 10 to surface set

supercharge: adding 12 to surface set

supercharge: adding 20 to surface set

supercharge: adding 23 to surface set

supercharge: adding 24 to surface set

supercharge: adding 25 to surface set

supercharge: adding 29 to surface set

supercharge: adding 31 to surface set

In AvNAPSA-mode, AvNAPSA values are output for RK/DE/NQ residues:

supercharge: residue: 2 heavy: 9 sidechain: 5 AvNAPSA_value: 112

supercharge: residue 18 is already negative

supercharge: residue 20 is already negative

supercharge: residue: 22 heavy: 8 sidechain: 5 AvNAPSA_value: 135

supercharge: residue: 25 heavy: 9 sidechain: 5 AvNAPSA_value: 69

supercharge: residue: 29 heavy: 11 sidechain: 5 AvNAPSA_value: 99

supercharge: residue 31 is already negative

supercharge: residue 33 is already negative

supercharge: residue 35 is already negative

supercharge: residue: 38 heavy: 8 sidechain: 5 AvNAPSA_value: 81

supercharge: residue: 40 heavy: 9 sidechain: 5 AvNAPSA_value: 108

supercharge: residue: 44 heavy: 9 sidechain: 5 AvNAPSA_value: 142

supercharge: residue: 51 heavy: 9 sidechain: 5 AvNAPSA_value: 76

supercharge: residue: 65 heavy: 9 sidechain: 5 AvNAPSA_value: 197

supercharge: residue: 69 heavy: 11 sidechain: 5 AvNAPSA_value: 109

output PDB name

number of charged residues

net charge

mutations

number of mutations

pymol selection of mutated residues:

supercharge: NAME: 2B3P_A_min_RK_-1.98_-1.65_0002.pdb

supercharge: 2B3P_A_min_RK_-1.98_-1.65_0002.pdb R=17, K=44, D=10, E=10

supercharge: 2B3P_A_min_RK_-1.98_-1.65_0002.pdb Net Charge = 41

supercharge: 2B3P_A_min_RK_-1.98_-1.65_0002.pdb Mutations: V11R, D21R, H25R, T38K, T43R, D76K, E95K, T97K, D102K, T105R, D117K, E124K, I128K, D129R, E132K, E142R, N149K, T153K, Q157K, N159K, N164K, E172K, D180K, Y182K, D190K, V193K, L194K, N198K, Q204K, V206K, S208R, N212K, T230R, H231K,

supercharge: 2B3P_A_min_RK_-1.98_-1.65_0002.pdb # of mutations: 34

supercharge: 2B3P_A_min_RK_-1.98_-1.65_0002.pdb select 11+21+25+38+43+76+95+97+102+105+117+124+128+129+132+142+149+153+157+159+164+172+180+182+190+193+194+198+204+206+208+212+230+231+

Energy comparison of mutated residues, or all residues:

supercharge: Subtracting design from native energies

supercharge: 2B3P_A_min.pdb 10 0.3626 0.2911 -0.3058 0.3239 -1.4283 0 -1.0630 0 0 0 0 0 0 0 0 0.7930 0 2.3999 1.0869 -1.2700

supercharge: 2B3P_A_min.pdb 20 0.8852 0.6912 0.0107 -1.7965 0.7626 0 0.4028 0 0 0.0300 0 0 0 0 0 0.6864 0 2.5038 0.0948 -0.3100

supercharge: 2B3P_A_min.pdb 24 -0.1093 0.0870 0.0103 0.5427 1.0906 0 -0.0248 0 0 -0.0654 0 0 0 0 0 0.4718 0 1.3747 0.6997 -1.5400

supercharge: 2B3P_A_min.pdb 37 -0.0362 0.0129 0.0006 -0.2910 -1.0171 0 0.2086 0 0 0 0 0 0 0 0 -0.8536 0 1.3987 -0.5885 -0.3800

supercharge: 2B3P_A_min.pdb 42 0.2946 -0.2654 0.0031 0.0717 1.0044 0 -0.4215 0 0 0 -0.1038 0 0 0 0 -0.6141 0 2.9814 -0.1912 -0.7100

supercharge: 2B3P_A_min.pdb 72 0.3975 0.2649 -0.0007 -0.5445 0.0098 0 0.3601 0 0 0 0 0 0 0 0 -0.4753 0 0.9331 -0.2628 0.0200

supercharge: 2B3P_A_min.pdb 91 0.1133 -0.2226 0.1196 -0.0862 -0.5008 0 0.5076 0 0 0 0 0 0 0 0 -0.2621 0 -0.1621 0.0978 0.1600

supercharge: 2B3P_A_min.pdb 93 0.5722 0.1466 -0.0032 -0.1445 -0.4706 0 -0.2336 0 0 0 0 0 0 0 0 0.1144 0 1.4960 0.5811 -0.3800

supercharge: 2B3P_A_min.pdb 98 0.0845 0.1706 0.1538 -0.7029 0.2191 0 0.4510 0 0 0 0 0 0 0 0 0.1411 0 -0.0224 0.2489 0.0200

supercharge: 2B3P_A_min.pdb 101 0.6640 -0.3440 0.1201 0.2472 0.0273 0 -0.1159 0 0 0 -0.0153 0 0 0 0 -0.2019 0 2.5720 0.3413 -0.7100

supercharge: 2B3P_A_min.pdb 113 0.1539 0.5641 -0.0004 -0.9042 -1.4567 0 0.1523 0 0 0 0.0339 0 0 0 0 0.2621 0 -0.0121 0.3713 0.0200

supercharge: 2B3P_A_min.pdb 120 0.0583 -0.0026 0.0007 -0.0908 -0.4659 0 0.1072 0 0 0 0 0 0 0 0 -0.0543 0 -0.1800 0.0626 0.1600

supercharge: 2B3P_A_min.pdb 124 -0.0042 1.2284 -0.0227 -1.0185 -3.5904 0 -0.1822 0 0 0 0 0 0 0 0 0.0013 0 1.0539 0.2759 -0.8900

supercharge: 2B3P_A_min.pdb 125 0.2091 0.5483 0.0073 -0.7297 0.9577 0 0.0954 0 0 0 0 0 0 0 0 0.3621 0 0.5631 0.3540 -0.3100

supercharge: 2B3P_A_min.pdb 128 0.0433 -0.0009 0.0004 0.0062 -0.3876 0 0.0020 0 0 0 0 0 0 0 0 -0.0305 0 -0.0963 -0.1860 0.1600

supercharge: 2B3P_A_min.pdb 138 0.1440 0.4522 -0.0416 -0.9915 0.6102 0 0.1466 0 0 0 0.0668 0 0 0 0 -0.0257 0 0.8851 -0.0726 -0.1700

supercharge: 2B3P_A_min.pdb 145 -0.3478 0.0592 -0.0288 -0.9271 0.0888 0 0.1110 0 0 0 0.0067 0 0 0 0 -0.6491 0 0.4166 -0.5798 0.2400

supercharge: 2B3P_A_min.pdb 149 0.1701 -0.2459 -0.0052 -0.0518 -1.1845 0 -0.1135 0 0 0.0633 0 0 0 0 0 0.5776 0 0.7759 0.6844 -0.3800

supercharge: 2B3P_A_min.pdb 153 -0.2278 -0.2601 0.0002 -0.1440 0.0400 0 0.1631 0 0 0 0 0 0 0 0 -0.4850 0 -0.3687 -0.0711 0.3200

supercharge: 2B3P_A_min.pdb 155 0.5407 0.3020 0.1436 -0.6233 0.0553 0 0 0 0 0 0 0 0 0 0 1.0035 0 -0.4034 1.3313 0.2400

supercharge: 2B3P_A_min.pdb 160 -0.0473 0.3212 0.0081 -0.6022 0.0747 0 -0.0138 0 0 0 0 0 0 0 0 -0.7511 0 0.3574 -0.6246 0.2400

supercharge: 2B3P_A_min.pdb 168 -0.3021 0.0274 0.0131 -0.6476 -0.5254 0 0.1083 0 0 0 0 0 0 0 0 0.2500 0 -0.4034 0.1755 0.1600

supercharge: 2B3P_A_min.pdb 176 0.8351 1.8314 -0.3253 -2.2487 -1.4861 0 0.2085 0 0 0 0.0336 0 0 0 0 0.7663 0 0.6481 0.9543 0.0200

supercharge: 2B3P_A_min.pdb 178 -0.3683 2.0373 -0.3322 -1.8179 -4.8053 0 -0.6891 0 0 0 0.0336 0 0 0 0 0.4646 0 0.9837 0.5180 -1.1600

supercharge: 2B3P_A_min.pdb 186 0.5944 0.0793 0.0447 -0.1092 -0.1395 0 0.1015 0 0 0 0 0 0 0 0 -0.1487 0 0.9292 0.0703 0.0200

supercharge: 2B3P_A_min.pdb 189 -0.1863 0.3694 -0.0074 -0.0359 -2.3352 0 -0.2577 0 0 0 0 0 0 0 0 -0.7215 0 1.3672 -0.0030 -0.9400

supercharge: 2B3P_A_min.pdb 190 0.3781 0.3658 -0.0074 -0.0401 -1.1546 0 -0.0018 0 0 0 0 0 0 0 0 -0.0511 0 0.9868 0.3996 -0.5500

supercharge: 2B3P_A_min.pdb 194 -0.5029 0.6575 -0.0044 -1.3504 -0.5447 0 0.0584 0 0 0 0 0 0 0 0 -0.5862 0 -0.3094 -0.3911 0.2400

supercharge: 2B3P_A_min.pdb 200 0.2763 0.1326 -0.0011 -0.1518 -0.1089 0 0.0154 0 0 0 0 0 0 0 0 -0.1132 0 -0.1193 0.0992 0.3200

supercharge: 2B3P_A_min.pdb 202 0.6845 0.2154 -0.0597 -0.0909 -1.7388 0 0.0148 0 0 0 0 0 0 0 0 1.0054 0 1.5020 1.5470 -0.9400

supercharge: 2B3P_A_min.pdb 204 0.7408 -0.5101 0.1792 -0.2004 2.0877 0 0.0232 0 0 0 0 0 0 0 0 0.7133 0 2.1510 1.3854 -0.6100

supercharge: 2B3P_A_min.pdb 208 0.5157 0.3552 0.0041 -0.6123 -0.0296 0 0.1402 0 0 0 0 0 0 0 0 0.6437 0 -0.1138 0.7940 0.2400

supercharge: 2B3P_A_min.pdb 226 0.7089 0.1036 0.0097 -0.2365 0.6538 0 0.0037 0 0 0 0 0 0 0 0 0.9292 0 1.5962 1.2544 -0.7100

supercharge: 2B3P_A_min.pdb 227 -0.7347 0.9993 -0.0509 -0.7053 -0.0501 0 0.0871 0 0 0 0 0 0 0 0 -0.0760 0 0.2680 -0.0646 -1.2100

supercharge: 2B3P_A_min.pdb SUM-DIFFS 6.5603 10.4623 -0.3673 -16.7038 -15.7384 0 0.3517 0 0 0.0279 0.0556 0 0 0 0 3.0867 0 27.9528 10.3922 -10.5900

score_terms: total fa_atr fa_rep fa_sol fa_intra_rep pro_close fa_pair hbond_sr_bb hbond_lr_bb hbond_bb_sc hbond_sc dslf_ss_dst dslf_cs_ang dslf_ss_dih dslf_ca_dih rama omega fa_dun p_aa_pp ref

These energies will be slightly different between runs because the packing algorithm is stochastic. However, for repacking a protein surface, the energies are likely to be very similar. Multiple runs (<10 should suffice) will help you gauge how well the packing converges.

Output resfile: In addition to the optional input resfile, this resfile is generated by the supercharge protocol and governs the design run. It is provided as output for the user’s information.

NATAA

start

6 A PIKAA ERK

9 A PIKAA TRK

11 A PIKAA VRK

21 A PIKAA DRK

25 A PIKAA HRK

30 A NATAA #same charge

32 A NATRO #has sc hbond energy=-1.15844

38 A PIKAA TRK

39 A NATRO #has sc hbond energy=-1.33149

43 A PIKAA TRK

52 A NATAA #same charge

76 A PIKAA DRK

77 A PIKAA HRK

80 A NATAA #same charge

90 A NATRO #has sc hbond energy=-1.15595

**Gene synthesis, cloning, and expression of charged GFP variants**:

Genes for each GFP variant were synthesized from 100 nucleotide oligos assembled by PCR. Assembled genes were then cloned into pET21 and verified by sequencing. Variants RSC10 and RSC16 showed significant sequence errors, but the remaining verified clones were transformed into BL21 (DE3) cells. Individual colonies were picked from each plate and inoculated into 2 ml auto-induction media. Cultures were grown for 24 hours at 37^o^C. Each culture was scanned in a Tecan Safire plate reader at wavelength 600 nm, and 1 ml aliquots of each culture were normalized according to absorbance at 600 nm. 500 μl aliquots from each normalized culture were spun down at 2,000xg for 20 minutes at 4^o^C and resuspended in 500 μl PBS. Cells were then sonicated at 20% amplitude for 20 seconds with a 50% duty cycle. Lysates were cleared by centrifugation at 10,000xg for 30 minutes at 4^o^C. Each cleared lysate was then scanned at emission/excitation wavelengths of 395/509 nm with 10 nm slit (**Figure 4**). The insoluble pellet from the above was saved and resuspended in 500 μl 5M NaCl. Each high salt lysate was then scanned at 395/509 nm with a 10 nm slit.
